# *Toxoplasma gondii* from Gabonese forest, Central Africa: First report of an African wild strain

**DOI:** 10.1371/journal.pntd.0012214

**Published:** 2025-01-06

**Authors:** Lokman Galal, Matthieu Fritz, Pierre Becquart, Karine Passebosc-Faure, Nicolas Plault, Larson Boundenga, Illich Manfred Mombo, Linda Bohou Kombila, Telstar Ndong Mebaley, Léadisaelle Hosanna Lenguiya, Barthélémy Ngoubangoye, Nadine N’Dilimabaka, Franck Prugnolle, Lionel Forestier, Endrias Zewdu Gebremedhin, Eric M. Leroy, Gael Darren Maganga, Aurélien Mercier

**Affiliations:** 1 Inserm U1094, IRD UMR270, Univ. Limoges, CHU Limoges, EpiMaCT - Epidemiology of Chronic Diseases in Tropical Zone, Institute of Epidemiology and Tropical Neurology, OmegaHealth, Limoges, France; 2 Maladies Infectieuses et Vecteurs, Ecologie, Génétique, Evolution et Contrôle (MIVEGEC), University of Montpellier, IRD, CNRS, Montpellier, France; 3 Centre National de Référence (CNR) Toxoplasmose/Toxoplasma Biological Center (BRC), Centre Hospitalier-Universitaire Dupuytren, Limoges, France; 4 Centre Interdisciplinaire de Recherches Médicales de Franceville (CIRMF), Franceville, Gabon; 5 Department of Anthropology, University of Durham, Durham, United Kingdom; 6 Faculté des Sciences et Techniques, Université Marien Ngouabi, Brazzaville, République du Congo; 7 CNRS, Laboratoire de Biométrie et Biologie Evolutive UMR5558, Université de Lyon 1, Villeurbanne, France; 8 Département de Biologie, Faculté des Sciences, Université des Sciences et Techniques de Masuku (USTM), Franceville, Gabon; 9 International Research Laboratory-REHABS, CNRS-Université Lyon 1-Nelson Mandela University, Nelson Mandela University George Campus, George, South Africa; 10 Department of Veterinary Science, School of Veterinary Medicine, Ambo University, Ambo, Ethiopia; 11 INSAB, Université des Sciences et Techniques de Masuku (USTM), Franceville, Gabon; Centro de Pesquisa Gonçalo Moniz-FIOCRUZ/BA, BRAZIL

## Abstract

The protozoan *Toxoplasma gondii* is a ubiquitous and highly prevalent parasite that can theoretically infect all warm-blooded vertebrates. In humans, toxoplasmosis causes infections in both immunodeficient and immunocompetent patients, congenital toxoplasmosis, and ocular lesions. These manifestations have different degrees of severity. Clinical severity is determined by multiple factors, including the genotype of the *T. gondii* strain involved in the infection. *T. gondii* exhibits remarkable genetic diversity, which varies according to geography and ecotype (domestic or wild). Previous studies have demonstrated that wild strains of *T. gondii* are of particular epidemiological interest, as they have been associated with more severe forms of toxoplasmosis in different regions of the world. However, no data on wild strains of *T. gondii* are available from Africa. In this study, we describe for the first time a wild *T. gondii* strain from Africa. Wild animals from the forest environment of Gabon, Central Africa, were screened for chronic infection with *T. gondii* using quantitative PCR. The infecting *T. gondii* strains were genotyped whenever possible by the analysis of 15 microsatellite markers and by whole-genome sequencing. A new *T. gondii* genotype was identified in the DNA extract from a heart sample of a duiker (*Cephalophus* sp.) and was found to be highly divergent from previously described *T. gondii* populations worldwide, including those from domestic environments in Gabon. This discovery suggests the existence of a wild *T. gondii* population in Africa. The role of wild *T. gondii* strains in the incidence of severe toxoplasmosis in Africa remains unclear and requires further investigation.

## Author summary

The emergence of new pathogens from wildlife is today a well-recognized health threat. Studying these infectious agents has proven to be challenging due to the difficulty in accessing samples from wild animals. In the present study, we took advantage of a recent survey on the viral carriage of wild animals from Gabon, Central Africa, to screen animal samples for the presence of the zoonotic protozoan *Toxoplasma gondii*. This ubiquitous and highly prevalent parasite can theoretically infect all warm-blooded vertebrates, including humans. This parasite is the etiological agent of toxoplasmosis, a disease causing a substantial public health burden worldwide through different clinical manifestations and varying degrees of severity. A novel genotype was identified in a wild antelope from the Gabonese forest, and was found to be highly divergent from previously described *T. gondii* populations worldwide, including those from the domestic environment in Gabon. This discovery suggests the existence of a wild *T. gondii* population in Africa. It has been shown that wild strains of *T. gondii* are of significant epidemiological relevance, as they have been associated with more severe forms of toxoplasmosis in different regions of the world. The implications of wild *T. gondii* strains in the incidence of severe toxoplasmosis in Africa remain unclear and merit further investigation.

## Introduction

*Toxoplasma gondii* is a ubiquitous zoonotic protozoan infecting all warm-blooded species, including humans. All these species can act as intermediate hosts for *T. gondii* by developing persistent tissue cysts after feeding from tissues of another infected intermediate host or following the ingestion of sporulated oocysts found in the environment [1]. These oocysts are excreted in the environment through the feces of members of the Felidae family, the only definitive hosts of this parasite, following their feeding on infected prey [2,3]. The oocysts sporulate within a few days following their excretion to become infective.

*T. gondii* is estimated to infect around 30% of the human population and is the etiological agent of toxoplasmosis, a disease causing a substantial public health burden worldwide [4]. Historically, infection with *T. gondii* has been long considered essentially asymptomatic or benign, except for certain risk groups, such as the developing fetus in the case of congenital infection, and immunocompromised patients for whom toxoplasmosis can have severe health consequences either during primo-infection or reactivation [5]. However, clinical toxoplasmosis has also been reported in immunocompetent individuals, mainly in the form of ocular lesions and multi-visceral involvement [6–8]. The prevalence of clinical toxoplasmosis, its clinical forms and their severity substantially vary worldwide [9]. *T. gondii* strains diversity, which exhibits contrasting patterns across geographic regions and ecotypes, appears to explain, at least in part, this clinical variability [10].

In Africa, the few available reports of clinical toxoplasmosis in immunocompetent individuals suggest that certain *T. gondii* strains on this continent are more pathogenic than European strains [11–15]. However, the association between genotype and disease severity is still unclear due to the scarcity of reports. Furthermore, nearly all *T. gondii* isolates have been collected on human and domestic animals, and no sylvatic cycle of *T. gondii* has been described on this continent. Wild strains of *T. gondii*—defined as strains isolated from wild animals or from humans in contact with wildlife and genetically distinct from *T. gondii* populations found in the domestic environment—have been demonstrated to hold significant epidemiological importance, as they have been associated with more severe forms of toxoplasmosis in other regions of the world [16–18]. The objective of this study was to provide the first insights into the *T. gondii* strains circulating in African wildlife and to provide evidence for the existence of a sylvatic cycle of *T. gondii* in Africa involving genetically distinct *T. gondii* strains.

## Materials and methods

### Ethics statement

For Gabonese samples, authorization to capture and collect animals was obtained from the Ministry of Water and Forests, in charge of the environment and sustainable development (Authorization No. 0247 MEFCEDD/SG/DGFAP) [19]. For Ethiopian sample, the research project was approved by the animal ethical committee of the College of Veterinary Medicine and Agriculture, Addis Ababa University. All efforts were made to minimize animal suffering during the course of the study [20].

**Fig 1:**
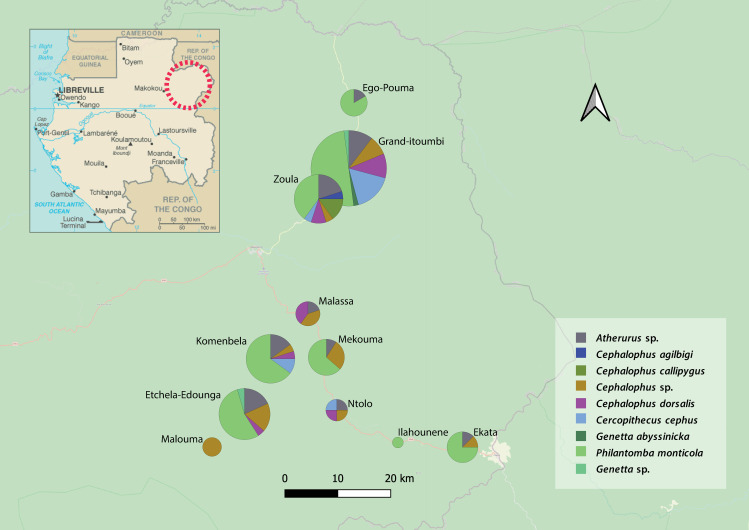
Map of the study area, Zadié Department, Gabon. In the country-wide map of Gabon (upper left), the study area is surrounded by a dotted red circle. Each pie chart represents a village where hunters brought back and sold bushmeat. The sizes of the pie charts correlate with the total number of animals sampled for each village, and the colors indicate different animal species. Map data OpenStreetMap contributors, licensed under ODbL (https://www.openstreetmap.org/copyright).

In the present study, we took advantage of a recent survey on the viral carriage of wild animals from northeast Gabon, central Africa [19]. Organ samples (brain, heart, lung and kidney) were previously collected from wild animals hunted in the surrounding forest of 11 villages (Fig 1) in the department of Zadié, province of Ogooué-Ivindo, in northeast Gabon in 2019 [19]. The 148 animals included in this study were of at least seven distinct wild mammal species (Fig 1). However, the number could have been underestimated given that only the genus could be determined for 43 animals. All the four organs considered in this study (brain, heart, lung and kidney) were available—and therefore were tested using quantitative polymerase chain reaction (qPCR)—in 80 animals while between one and three organs were available and were tested in the remaining 68 animals (S1 Table). No signs of disease (e.g., lesions or abnormal appearance) were detected on the animal’s organs. Here, these organs were processed and analyzed individually in order to detect chronic *T. gondii* infection in these animals. For this purpose, around 30 mg of each organ was collected when available and transferred to Lysing Matrix E tubes (MP Biomedicals) at −80 °C until processing. Samples were mechanically disrupted using a TissueLyser II (Qiagen, Courtaboeuf, France) for 30 s at 30 Hz. Then, cooling of samples was performed in dry ice for 45 s before carrying out the second round of mechanical disruption under the same conditions. Tubes were then centrifugated at 200 × g for 5 min and 350 µL of lysate was collected from each tube for DNA extraction using the QIAamp DNA Mini Kit (Qiagen, Courtaboeuf, France).

The extracted DNA samples were tested by qPCR assay as described by Ajzenberg et al. [21] on a thermocycler Rotor-Gene 6000 (Corbett Life Science, Sydney, Australia), targeting the 529 bp DNA fragment (REP529, GenBank accession no. AF146527 [22]). In brief, each PCR contained 5 μL of extracted DNA from organs, mixed with 15 μL of a PCR mix with 1X LightCycler FastStart DNA Master Hybridisation Probes kit (Roche Diagnostics, Mannheim, Germany), 0.5 U of UDG (Roche Diagnostics, Mannheim, Germany), 5 mmol/L of MgCl2, 0.5 μmol/L of each primer, 0.1 μmol/L of TaqMan probe (Eurofins, Ebersberg, Germany) which is labeled with a fluorescent dye (6-carboxyfluorescein, 6-FAM) at 5’ end and a dark quencher (Black Hole Quencher, BHQ1) at the 3’ end. The cycling protocol was as follows: initial decontamination by UDG at 50 °C for 2 min and denaturation at 95 °C for 10 min, followed by 50 cycles at 95 °C for 20 s and 60 °C for 40 s. Each sample was run in duplicate and the results obtained were expressed in cycle threshold (Ct) values.

*T. gondii* strains were genotyped using 15 microsatellite (MS) markers distributed across 11 of the 13 chromosomes composing the *T. gondii* genome in a single multiplex PCR assay as described previously [23], following the guidelines established by Joeres et al. [24]. Those 15 loci included a combination of eight “typing” markers with low polymorphism (TUB2, W35, TgM-A, B17, B18, M33, IV.1 and XI.1) that show little or no variation within lineages and seven “fingerprinting” markers (M48, M102, N83, N82, AA, N61, N60) exhibiting high polymorphism and significant variation within lineages. PCR products were sized using capillary electrophoresis on ABI PRISM 3130xl (Applied Biosystems, Foster City, CA) and the GenScan 500 ROX dye size standard (Applied Biosystems). Results were analysed using GeneMapper 5.0 software packages (Applied Biosystems). New multilocus genotypes (MLGs) were compared to those from a global dataset (S1 Table) of previously published MLGs (n = 1059) by generating a neighbor-joining dendrogram using the BRUVO.BOOT function (based on Bruvo’s genetic distance) with 1,000 bootstrap replications, as implemented in the “Poppr” package [24] in R version 3.4.0. In addition, the factorial correspondence analysis (AFC) technique available in GENETIX version 4.05 [25] was used to visualize the genetic distance between MLGs in a multidimensional space (3D).

As a complement to MS genotyping, whole genome sequencing (WGS) was performed on successfully genotyped DNA samples. These DNA samples were subjected to high-throughput sequencing (HTS) on the Illumina NovaSeq 6000 platform (Novogene, United Kingdom). For comparison purposes, WGS was also performed on a DNA sample of the Ethiopian *T. gondii* strain TgSpEt19, extracted from mouse ascites obtained during strain bioassay [20]. This DNA sample was subjected to HTS on the Illumina NextSeq 500 sequencing device of the BISCEm technical and bioinformatics platform at the University of Limoges (BISCEm University of Limoges/ US 42 INSERM/ UAR 2015 CNRS). FastQC was applied to analyze read quality and adapters were trimmed with Trimmomatic v0.39 to truncate low-quality reads, filtering for a minimum read length of 36 (parameters: SLIDINGWINDOW: 4:20; MINLEN: 36; TruSeq3-PE-2.fa:2:30:10; HEADCROP:10). Low-complexity sequences were filtered using Prinseq with the DUST method (lc_method = dust) and a threshold of 7 (lc_threshold = 7) to remove sequences with repetitive patterns or low complexity. Additionally, duplicate reads were identified and removed if they contained 14 or more consecutive identical base pairs (derep = 14) to reduce redundancy and minimize amplification bias. The genomic relatedness between new genomes and global *T. gondii* haplogroups (hg) was assessed through mapping the reads of each new genome against 16 *T. gondii* reference genomes representing 15 of the 16 *T. gondii* hg previously described worldwide (two reference genomes of the 6th hg were included and the 16th hg was unavailable) [26]. Quality control of the 16 reference genomes included masking low-complexity regions using RepeatMasker 4.0.9, identifying, filtering out contigs shorter than 1000 bases and removing potential contaminant contigs through BLAST analysis. Following these quality control steps, mapping was performed using FastQ Screen software, configured to utilize the BWA-MEM2 alignment tool. FastQ Screen provides information on the number of reads that map exclusively against each of the selected reference genomes. Stringent parameterization of BWA-MEM2 (-T 100, -B 70, -O 30, -E 20) was applied to enhance alignment accuracy by reducing mismatches, minimizing gaps, and improving the reliability of read mapping to closely related reference genomes. To validate this method, FastQ Screen was first run on eight previously described genomes [27]. Once validated, the approach was applied to the new genomes from the present study.

## Results

*T. gondii* DNA was detected in 15 animals belonging to at least four distinct species (*Philantomba monticola*, *Cephalophus callipygus*, *Cephalophus dorsalis*, *Cephalophus* sp. and *Atherurus* sp.) and from six different villages (S1 Table).

Molecular prevalences among species ranged from 0% to 33.3% (S2 Table). In the 15 PCR-positive animals, one to three organ types were PCR-positive, while none were found to be PCR-positive for the four organs. For each PCR-positive animal, the organ sample showing the lower C_t_ value was selected for MS genotyping. MS markers were amplified in DNA extracted from (1) the heart of Gabon-87_2019-*Cephalophus*-sp (ID: 87), collected in Mekouma. The DNA sample had Ct values of 24.33 and 24.43, corresponding to *T. gondii* cell concentrations of 46 and 43 copies/µL, and 12 out of 15 MS markers were successfully amplified; and from (2) the brain of Gabon-21_2019-*Cephalophus-callipygus* (ID: 21), collected in Zoula. The DNA sample had Ct values of 38.16 and 37.81, corresponding to *T. gondii* cell concentrations below 1 copy/µL, and 1 out of 15 MS markers was successfully amplified. The two samples displayed a novel allele (228 at M102, a fingerprinting marker), not previously observed in any of the previously published MLGs (S1 Table), suggesting that they may belong to the same population. This allele was highly divergent from all other alleles previously reported for this MS marker, which displays allele sizes ranging from 164 to 196, except for TgSpEt19, a strain from a sheep in Ethiopia, which displayed a fragment of 218 at this marker. Gabon-87_2019-Cephalophus-sp exhibited another novel allele (154 at B18). Notably, it also shared four of its five amplified typing markers with TgSpEt19 (Table 1).

**Table 1:** *Toxoplasma gondii* microsatellite analysis of organ samples from Gabonese wild animals and a comparison with other Gabonese, African and global isolates. New MS alleles are indicated in bold letters.

Strain ID	Origin	Host	Type or lineage	Typing markers								Fingerprinting markers							
				TUB2	W35	TgM-A	B18	B17	M33	IV.1	XI.1	M48	M102	N60	N82	AA	N61	N83	Reference
Gabon-87_2019-*Cephalophus*-sp	Gabon	*Cephalophus* sp.	Unclassified genotype	289	240	201	**154**	340	ND	ND	ND	209	**228**	138	109	287	97	333	This study
Gabon-21_2019-*Cephalophus*-callipygus	Gabon	*Cephalophus callipygus*	Unclassified genotype	ND	ND	ND	ND	ND	ND	ND	ND	ND	**228**	ND	ND	ND	ND	ND	This study
GAB5-2007-GAL-DOM1	Gabon	*Gallus domesticus*	Africa 1	291	248	205	160	342	165	274	354	231	166	149	111	277	87	306	[28]
GAB1-2007-GAL-DOM10	Gabon	*Gallus domesticus*	Africa 3	291	242	207	160	342	165	278	354	225	166	142	111	275	97	310	[28]
GAB1-FEL-CAT001	Gabon	*Felis catis*	Type III	289	242	205	160	336	165	278	356	213	190	149	111	267	89	312	[28]
GAB2-2007-GAL-DOM006	Gabon	*Gallus domesticus*	unclassified genotype	289	242	207	160	336	165	278	356	223	190	147	111	261	103	316	[28]
GAB4-2007-GAL-DOM001	Gabon	Chicken *Gallus domesticus*	unclassified genotype	291	242	205	160	336	165	278	356	213	190	145	111	269	89	306	[28]
TgSpEt19	Ethiopia	O*vis aries*	Unclassified genotype	289	240	201	156	340	163	272	360	209	218	147	127	283	99	342	[20]
ENT	France	*Homo sapiens*	I	291	248	209	160	342	169	274	358	209	166	145	119	267	87	306	
ME49	USA	O*vis aries*	II	289	242	207	158	336	169	274	356	215	174	142	111	265	91	310	
NED	France	*Homo sapiens*	III	289	242	205	160	336	165	278	356	209	190	147	111	267	91	312	

**Fig 2:**
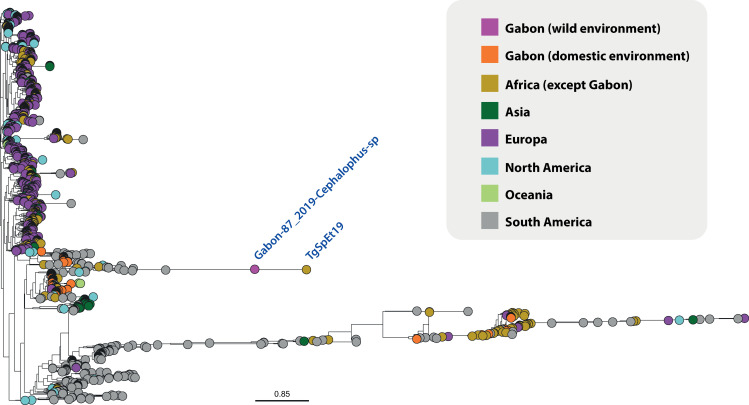
Neighbor-joining tree showing the relationships between Gabon-87_2019-*Cephalophus*-sp and other global multilocus genotypes (MLGs) (n = 1059) from previous studies.

**Fig 3:**
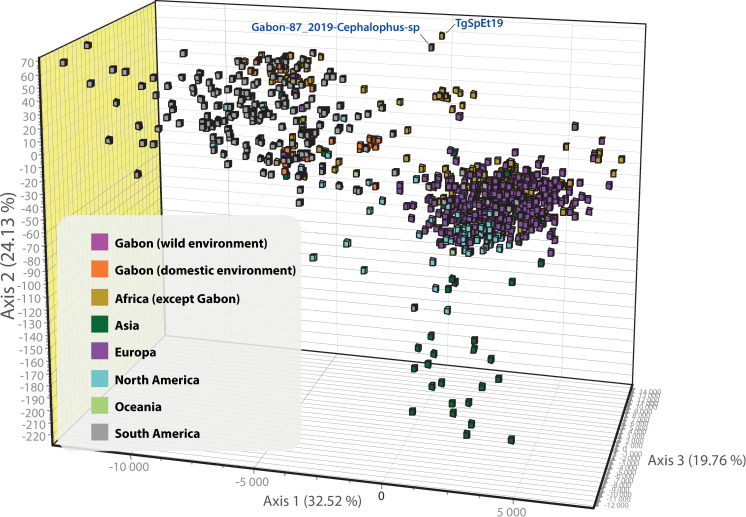
Factorial correspondence analysis (AFC) technique including Gabon-87_2019-*Cephalophus*-sp and other global multilocus genotypes (MLGs) (n = 1059) from previous studies.

The NJ dendrogram (Fig 2) and AFC (Fig 3)—based on the analysis of the 12 MS markers that were successfully amplified in Gabon-87_2019-*Cephalophus*-sp—confirmed that this sample was genetically related to TgSpEt19 and exhibited a divergent profile compared to other previously described MLGs worldwide (S3 Table), including those from Gabon’s domestic environment.

**Fig 4:**
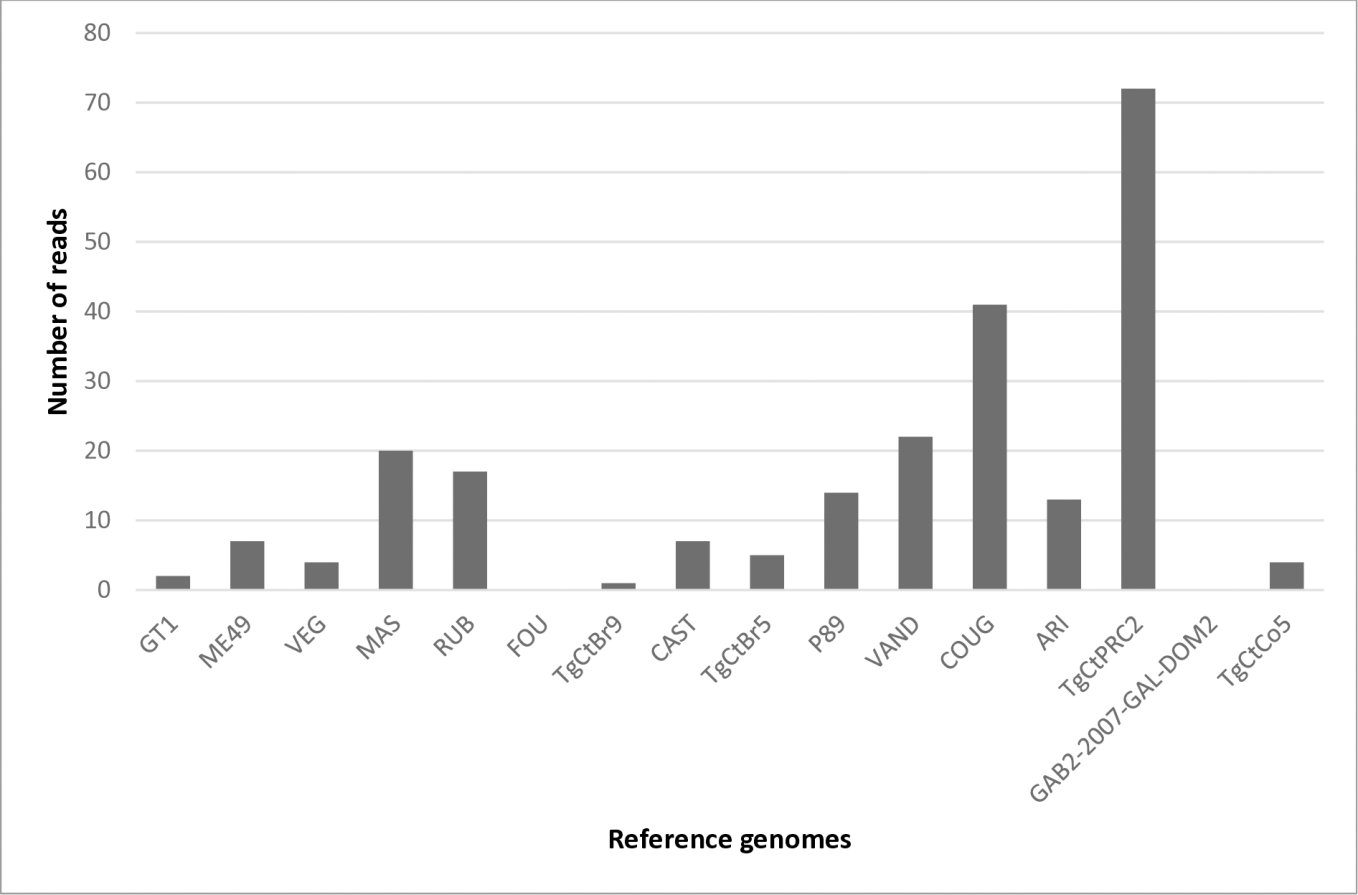
Comparison of *Toxoplasma gondii* genome Gabon-87_2019-*Cephalophus*-sp to reference genomes representing the global haplogroups. The barplots represent the numbers of Illumina reads exclusively mapping to each of the 16 respective reference genomes. These references genomes are GT1 (USA; type I; hg1; GCA_000149715.2), ME49 (USA; type II; hg2; GCA_000006565.2), VEG (USA; type III; hg3; GCA_000150015.2), MAS (unknown origin; hg4; GCA_000224865.2), RUB (French Guiana; Amazonian; hg5; GCA_000224805.2), FOU (unknown origin; Africa 1; hg6; GCA_000224905.2), TgCtBr9 (Brazil; hg6; GCA_000224825.1), CAST (USA; hg7; GCA_000256705.1), TgCtBr5 (Brazil; hg8; GCA_000259835.1), P89 (USA; hg9; GCA_000224885.2), VAND (French Guiana; Amazonian; hg10; GCA_000224845.2), COUG (Canada; Pan-American; hg11; GCA_000338675.1), ARI (USA; type 12; hg12; GCA_000250965.1), TgCtPRC2 (China; Chinese 1; hg13; GCA_000256725.1), GAB2-2007-GAL-DOM2 (Gabon; Africa 3; hg14; GCA_000325525.2) and TgCtCo5 (Colombia; hg15; GCA_000278365.1).

Among the 15 PCR-positive animals, only Gabon-87_2019-*Cephalophus*-sp (DNA from heart sample) was selected for Illumina WGS. A total of 1,301,636,628 paired-end reads (150 nt × 2) were obtained for this sample following quality control. Similarly, 218,855,336 paired-end reads (75 nt × 2) were obtained for the TgSpEt19 DNA sample after quality control. In parallel, quality control measures applied to the reference genomes enabled the identification and removal of contaminant contigs (e.g., host or bacterial sequences) in 13 out of 16 genomes (S4 Table). FastQ Screen produced meaningful results on the eight genomes previously selected for validating the approach (S1 Fig), and was subsequently applied to each of the two new genomes. A high proportion of *T. gondii* reads from the Gabon-87_2019-*Cephalophus*-sp sample mapped exclusively against TgCtPRC2 and COUG (Fig 4). No reads from TgSpEt19 mapped against any of the 16 reference genomes, likely due to the combination of stringent alignment settings and the shorter read length, which reduced the likelihood of achieving sufficient alignment scores for mapping. Gabon-87_2019-*Cephalophus*-sp sequencing data have been deposited in the European Nucleotide Archive (ENA) database under accession code ERR13964874.

## Discussion

This is the first study to explore *T. gondii* circulation in the wild environment of a tropical African country and to provide evidence for the existence of wild *T. gondii* strains in Africa. It also reports for the first time the presence of *T. gondii* in the tissues of species such as duikers (*Philantomba* sp. and *Cephalophus* sp.) and brush-tailed porcupines (*Atherurus* sp.). Molecular prevalence among species was relatively low but challenging to compare due to the small sample sizes for several species and the inaccurate identification of approximately one-third of the animals. In addition, the molecular prevalence levels may have been slightly overestimated due to the possible weak cross-reactivity of the primers targeting the *T. gondii* 529 bp repeat region with *Hammondia* DNA, as previously reported by Schares et al. [29].

To date, the presence of wild *T. gondii* populations could only be confirmed in North America and French Guiana, in South America [27,30–32]. These regions are characterized by the persistence of wild felid populations of relatively large sizes [33–35], which are probably the main drivers of the maintenance of *T. gondii* sylvatic cycles involving genetically distinct *T. gondii* strains. In other countries where representative sampling of humans, domestic and wild animals has been carried out, such as China and France, *T. gondii* clonal lineages isolated from all these host categories essentially belonged to the same domestic lineages (mainly Chinese 1 and type I in China and type II in France) [36–39]. In China and France, wild felid populations have undergone a significant decline due to the destruction of their habitats [40,41]. This situation could have resulted in the disappearance of sylvatic cycles of *T. gondii—*and *T. gondii* strains associated to these sylvatic cycles –in these countries, potentially explaining the lack of reports on wild *T. gondii* populations there. Furthermore, a recent study also indicates a lack of ecotype compartmentalization in *T. gondii* populations from Brazil [42], although the numerous gaps in sampling in this country—especially from wildlife—make it challenging to draw definitive conclusions at this stage. It is noteworthy that natural habitats in Brazil are subject to significant degradation in comparison to those neighboring French Guiana [43], which may explain the absence of ecotype compartmentalization. In Gabon, the equatorial forest is one of the few well-preserved ecosystems of its kind in Africa [44] and is a refuge for wild feline species such as the leopard (*Panthera pardus*) and the African golden cat (*Caracal aurata*). This situation may be conducive to the persistence of wild *T. gondii* populations in this ecosystem.

The various patterns of ecotype compartmentalization observed in *T. gondii* populations according to geographical areas have presented a challenge in the assignment of a *T. gondii* strain to an ecotype. In Africa, a few reports of *T. gondii* genotypes from wild animals have been documented, with *T. gondii* strains identified in an African francolin from Senegal and a jackal from South Africa [45,46]. However, these animals were infected with *T. gondii* strains commonly found in domestic environments, as seen in France and China, likely due to exposure to *T. gondii* from domestic sources (e.g., long-distance dissemination of cat oocysts or predation/scavenging on domestic animals).The most commonly accepted definition of a wild strain is that it is restricted to the wild environment and genetically distinct from strains found in the domestic environment within the same geographical region [27,31,42]. This is the first time a *T. gondii* strain from Africa fits this definition, as it was isolated from wildlife and was found to be highly divergent from *T. gondii* populations previously described in domestic animals in Gabon [22]. A recent study on *T. gondii* genomics identified a ~100-kb genomic region on chromosome 1a that has proven to be a robust marker for distinguishing *T. gondii* strains from different ecotypes (domestic or wild). This study showed that, on a global scale, *T. gondii* strains from the domestic environment display a unique haplotype in this genomic region on chromosome 1a, which is considered a probable specific adaptation to domestic cats. Conversely, wild *T. gondii* strains exhibit a high diversity of haplotypes at this genomic locus [27]. In this instance, the haplotype carried by Gabon-87_2019-*Cephalophus*-sp could not be characterized due to the low proportion of *T. gondii* DNA in the sample.

Studies on the genetic diversity of *T. gondii* have revealed significant diversity that can only be effectively captured through multilocus genotyping methods, such as MS analysis, restriction fragment length polymorphism (RFLP), and multilocus sequence typing (MLST) [47]. Among these, MS genotyping offers higher resolution compared to RFLP and benefits from the availability of numerous MS genotypes published worldwide, making it more advantageous for comparative studies than MLST, despite the latter’s higher sensitivity. While WGS offers an even more comprehensive approach, its implementation remains more challenging. In the present study, the MS-based analyses revealed a marked genetic proximity between the wild Gabonese *T. gondii* strain Gabon-87_2019-*Cephalophus*-sp and a unique strain isolated from a sheep in Ethiopia [20]. The flock this sheep belonged to had a grazing area commonly frequented by several species of wild felids [48], which could have been the source of contamination of this sheep. A similar pattern is observed in North America, where grazing domestic animals are substantially more exposed to wild *T. gondii* strains than farm-bound animals [32]. This genetic proximity between Ethiopian and Gabonese strains reveals that certain branches of the *T. gondii* evolutionary tree remain obscure. The global proliferation of domestic cats has favored the spread of a few cat-adapted clonal lineages [27], which have probably overwhelmed ancient *T. gondii* populations in many regions of the globe. The massive decline of most wild felid populations has likely confined wild *T. gondii* strains to a few relatively well-preserved ecosystems (refuge zones) where sizable wild felid populations are still maintained, as observed in French Guiana and North America [34,49]. This situation has probably facilitated the sampling of wild *T. gondii* strains in these two latter regions. In contrast, wild *T. gondii* strains transmitted by African, European and Asian wild felids remain almost unknown [50]. The inclusion of wild *T. gondii* strains from these regions in phylogenetic analyses could challenge the current paradigm of a South American origin for current *T. gondii* populations [51]. This is particularly relevant given the probable origin of the Felidae family in Asia [52].

A whole genome-based comparison of Gabon-87_2019-Cephalophus-sp with reference genomes representing the major *T. gondii* hg worldwide indicated that this strain is more closely related to TgCtPRC2 and COUG than to the predominant clonal lineages found in Gabon and Africa (type II, Africa 1, and Africa 3). The TgCtPRC2 strain (also known as CHINA01), isolated from a domestic cat in China, belongs to the Chinese 1 lineage, a common lineage in East Asia [39], which is derived from the Africa 4 lineage—a lineage widespread across both Africa and Asia [10]. The comparison of Gabon-87_2019-Cephalophus-sp with Africa 4 could have provided valuable insights; however, no Africa 4 reference genome was available for this analysis. COUG strain (also designated as CANADA01) has been isolated from a cougar during the investigation of a large community outbreak of waterborne toxoplasmosis in humans in Canada. However, the involvement of this strain in human cases could not be demonstrated [53]. COUG strain belongs to a wild *T. gondii* population designated as Pan-American. In addition to Canada, *T. gondii* strains of the same population have been isolated from wild animals in the United States [54], Mexico [55] and French Guiana [31]. It is noteworthy that Pan-American population, Chinese 1 and Africa 4 lineages are strongly related as previously shown by Su et al. [56].

This relative genetic proximity between Gabon-87_2019-*Cephalophus*-sp on the one hand, and TgCtPRC2 and COUG on the other, could reflect ancient divergences between wild populations of *T. gondii*, likely associated with the historical global dissemination of wild felids. This observation further highlights the importance of exploring *T. gondii* strain diversity in Asia, which is likely the missing link in the ancient spread of ancestral *T. gondii* strains. However, while the whole-genome approach used in this study provided valuable insights into the relative genomic relatedness between the new *T. gondii* genomes and global *T. gondii* haplogroups, it did not offer precise phylogenetic positioning or ancestry characterization of the new genomes. The present approach therefore appears relevant only in the context of samples with low parasitic DNA, as is the case with most clinical, animal, or environmental samples. Only the isolation of live *T. gondii* strains from these wild populations could enable in-depth genomic analyses, but it has proven logistically challenging in remote, difficult-to-access areas.

In South and North America, it has been previously shown that *T. gondii* strains from wildlife—in addition to certain wild-derived *T. gondii* strains—are often associated with cases and outbreaks of severe ocular and/or systemic disease and unusual presentations of toxoplasmosis in immunocompetent patients [57–60]. These clinical forms have been increasingly diagnosed in the last two decades and are still considered to be underdiagnosed on these two continents. The present study provides evidence for the existence of a sylvatic cycle of *T. gondii* in Africa. The involvement of African wild *T. gondii* strains in the incidence of severe toxoplasmosis among immunocompetent individuals [11–13,15] requires further investigation.

## Supporting information

S1 Fig Validation of genetic classification of *Toxoplasma gondii* genomes with FastQ Screen. Eight *T. gondii* genomes of known phylogenetic classification and genomic ancestry composition [27] were utilized to validate the utilization of FastQ Screen for the genetic classification of genomes through mapping of their reads against as set of 16 reference genomes representing global *T. gondii* haplogroups. The barplots represent the numbers of Illumina reads from each of the eight *T. gondii* genomes exclusively mapping to each of the 16 respective reference genomes. *T. gondii* reads from (a) PORTUGAL10 (type I) mainly mapped against GT1 reference genome (USA; type I; hg1; GCA_000149715.2), those from (b) FRANCE01 (type II) mainly mapped against ME49 (USA; type II; hg2; GCA_000006565.2), those from (c) PORTUGAL09 (type III) mainly mapped against VEG (USA; type III; hg3; GCA_000150015.2), those from (d) BENIN01 (Africa 1) mainly mapped against FOU (unknown origin; Africa 1; hg6; GCA_000224905.2), those from (e) SENEGAL04 (Africa 4) mainly mapped against TgCtPRC2 (China; Chinese 1; hg13; GCA_000256725.1), which is mainly composed of Africa 4 ancestry composition [27], those from (f) FRENCHGUIANA08 (Amazonian) mainly mapped against RUB (French Guiana; Amazonian; hg5; GCA_000224805.2) and VAND (French Guiana; Amazonian; hg10; GCA_000224845.2), those from (g) FRENCHGUIANA10 (Pan-American) mainly mapped against COUG (Canada; Pan-American; hg11; GCA_000338675.1) and those from (h) MARTINIQUE01 (hybrid of Pan-American, type II and III) mainly mapped against COUG (Canada; Pan-American; hg11; GCA_000338675.1).
(DOCX)

S1 Table Detailed information on the molecular detection of *Toxoplasma gondii* in different organ samples from each individual animal. (XLSX)

S2 Table Molecular prevalence of *Toxoplasma gondii* by species. (XLSX)

S3 Table Global dataset of previously published *Toxoplasma gondii* Multilocus genotypes (MLGs) (n = 1059) obtained from the analysis of 15 microsatellite markers and used for comparison with new MLGs from this study. (XLSX)

S4 Table Comparative metrics following masking, size filtering, and contaminant removal across 16 reference genomes. (XLSX)

## References

[1] Dubey JP. Toxoplasmosis of animals and humans. 2nd ed. CRC Press. 2016.

[2] Frenkel JK, Dubey JP, Miller NL. *Toxoplasma gondii* in cats: fecal stages identified as coccidian oocysts. Science. 1970;167(3919):893–6. doi: 10.1126/science.167.3919.893 4903651

[3] Zhu S, Shapiro K, VanWormer E. Dynamics and epidemiology of *Toxoplasma gondii* oocyst shedding in domestic and wild felids. Transbound Emerg Dis. 2022;69(5):2412–23. doi: 10.1111/tbed.14197 34153160

[4] Montoya JG, Liesenfeld O. Toxoplasmosis. Lancet. 2004;363(9425):1965–76. doi: 10.1016/S0140-6736(04)16412-X 15194258

[5] Robert-Gangneux F, Dardé M-L. Epidemiology of and diagnostic strategies for toxoplasmosis. Clin Microbiol Rev. 2012;25(2):264–96. doi: 10.1128/CMR.05013-11 22491772 PMC3346298

[6] Cortés AD, Aguirre N. Severe disseminated acute toxoplasmosis in an adult immunocompetent patient from the Colombian Pacific. Biomedica. 2018;38(0):19–23. doi: 10.7705/biomedica.v38i0.4087 30184374

[7] Cortés DA, Aguilar MC, Ríos HA, Rodríguez FJ, Montes KV, Gómez-Marín JE, et al. Severe acute multi-systemic failure with bilateral ocular toxoplasmosis in immunocompetent patients from urban settings in Colombia. Am J Ophthalmol Case Rep. 2020;18:100661. doi: 10.1016/j.ajoc.2020.100661 32195446 PMC7078491

[8] Pena HFJ, Ferreira MN, Gennari SM, de Andrade HF Jr, Meireles LR, Galisteo AJ Jr. *Toxoplasma gondii* isolated from a Brazilian patient with rare pulmonary toxoplasmosis has a novel genotype and is closely related to Amazonian isolates. Parasitol Res. 2021;120(3):1109–13. doi: 10.1007/s00436-020-07008-4 33420622

[9] Gilbert RE, Freeman K, Lago EG, Bahia-Oliveira LMG, Tan HK, Wallon M, et al. Ocular sequelae of congenital toxoplasmosis in Brazil compared with Europe. PLoS Negl Trop Dis. 2008;2(8):e277. doi: 10.1371/journal.pntd.0000277 18698419 PMC2493041

[10] Galal L, Hamidović A, Dardé ML, Mercier M. Diversity of *Toxoplasma gondii* strains at the global level and its determinants. Food Waterborne Parasitol. 2019;15:e00052. doi: 10.1016/j.fawpar.2019.e00052 32095622 PMC7033991

[11] Beltrame A, Venturini S, Crichiutti G, Meroni V, Buonfrate D, Bassetti M. Recurrent seizures during acute acquired toxoplasmosis in an immunocompetent traveller returning from Africa. Infection. 2016;44(2):259–62. doi: 10.1007/s15010-015-0821-7 26168861

[12] Gachet B, Elbaz A, Boucher A, Robineau O, Fréalle E, Ajana F, et al. Acute toxoplasmosis in an immunocompetent traveller to Senegal. J Travel Med. 2018;25(1):tay086. doi: 10.1093/jtm/tay086 30247670

[13] Galal L, Ajzenberg D, Hamidović A, Durieux M-F, Dardé M-L, Mercier A. Toxoplasma and Africa: one parasite, two opposite population structures. Trends Parasitol. 2018;34(2):140–54. doi: 10.1016/j.pt.2017.10.010 29174610

[14] Leroy J, Houzé S, Dardé M-L, Yéra H, Rossi B, Delhaes L, et al. Severe toxoplasmosis imported from tropical Africa in immunocompetent patients: a case series. Travel Med Infect Dis. 2020;35:101509. doi: 10.1016/j.tmaid.2019.101509 31712179

[15] Artiaga A, Perez L, Pasquier G, Le Moing V. Toxoplasmose aiguë multiviscérale de l’immunocompétent: à propos d’un cas importé d’Afrique tropicale. Médecine et Maladies Infectieuses Formation. 2022;1:145–8.

[16] Carme B, Bissuel F, Ajzenberg D, Bouyne R, Aznar C, Demar M, et al. Severe acquired toxoplasmosis in immunocompetent adult patients in French Guiana. J Clin Microbiol. 2002;40(11):4037–44. doi: 10.1128/JCM.40.11.4037-4044.2002 12409371 PMC139686

[17] Demar M, Hommel D, Djossou F, Peneau C, Boukhari R, Louvel D, et al. Acute toxoplasmoses in immunocompetent patients hospitalized in an intensive care unit in French Guiana. Clin Microbiol and Infect. 2012;18(7):E221–31. doi: 10.1111/j.1469-0691.2011.03648.x 21958195

[18] Schumacher AC, Elbadawi LI, DeSalvo T, Straily A, Ajzenberg D, Letzer D, et al. Toxoplasmosis outbreak associated with *Toxoplasma gondii*-contaminated venison-high attack rate, unusual clinical presentation, and atypical genotype. Clin Infect Dis. 2021;72(9):1557–65. doi: 10.1093/cid/ciaa285 32412062 PMC9248295

[19] Becquart P, Bohou Kombila L, Mebaley TN, Paupy C, Garcia D, Nesi N, et al. Evidence for circulation of Rift valley fever virus in wildlife and domestic animals in a forest environment in Gabon, Central Africa. PLoS Negl Trop Dis. 2024;18(3):e0011756. doi: 10.1371/journal.pntd.0011756 38427694 PMC10936825

[20] Gebremedhin EZ, Abdurahaman M, Tessema TS, Tilahun G, Cox E, Goddeeris B, et al. Isolation and genotyping of viable *Toxoplasma gondii* from sheep and goats in Ethiopia destined for human consumption. Parasit Vectors. 2014;7:425. doi: 10.1186/1756-3305-7-425 25190185 PMC4161867

[21] Ajzenberg D, Lamaury I, Demar M, Vautrin C, Cabié A, Simon S, et al. Performance testing of PCR assay in blood samples for the diagnosis of toxoplasmic encephalitis in AIDS patients from the French departments of America and genetic diversity of *Toxoplasma gondii*: a prospective and multicentric study. PLoS Negl Trop Dis. 2016;10(6):e0004790. doi: 10.1371/journal.pntd.0004790 27355620 PMC4927177

[22] Homan WL, Vercammen M, De Braekeleer J, Verschueren H. Identification of a 200- to 300-fold repetitive 529 bp DNA fragment in *Toxoplasma gondii*, and its use for diagnostic and quantitative PCR. Int J Parasitol. 2000;30(1):69–75. doi: 10.1016/s0020-7519(99)00170-8 10675747

[23] Ajzenberg D, Collinet F, Mercier A, Vignoles P, Dardé M-L. Genotyping of *Toxoplasma gondii* isolates with 15 microsatellite markers in a single multiplex PCR assay. J Clin Microbiol. 2010;48(12):4641–5. doi: 10.1128/JCM.01152-10 20881166 PMC3008440

[24] Joeres M, Cardron G, Passebosc-Faure K, Plault N, Fernández-Escobar M, Hamilton CM, et al. A ring trial to harmonize *Toxoplasma gondii* microsatellite typing: comparative analysis of results and recommendations for optimization. Eur J Clin Microbiol Infect Dis. 2023;42(7):803–18. doi: 10.1007/s10096-023-04597-7 37093325 PMC10266996

[25] Belkhir K, Borsa P, Chikhi L, Raufaste N, Bonhomme F. GENETIX 4.05, logiciel sous Windows pour la génétique des populations. Montpellier, France: Laboratoire Génome, Populations, Interactions, CNRS UMR 5000. Université de Montpellier II. 2004.

[26] Lorenzi H, Khan A, Behnke MS, Namasivayam S, Swapna LS, Hadjithomas M, et al. Local admixture of amplified and diversified secreted pathogenesis determinants shapes mosaic *Toxoplasma gondii* genomes. Nat Commun. 2016;7:10147. doi: 10.1038/ncomms10147 26738725 PMC4729833

[27] Galal L, Ariey F, Gouilh MA, Dardé M-L, Hamidović A, Letourneur F, et al. A unique *Toxoplasma gondii* haplotype accompanied the global expansion of cats. Nat Commun. 2022;13(1):5778. doi: 10.1038/s41467-022-33556-7 36182919 PMC9526699

[28] Mercier A, Devillard S, Ngoubangoye B, Bonnabau H, Bañuls A-L, Durand P, et al. Additional haplogroups of *Toxoplasma gondii* out of Africa: population structure and mouse-virulence of strains from gabon. PLoS Negl Trop Dis. 2010;4(11):e876. doi: 10.1371/journal.pntd.0000876 21072237 PMC2970538

[29] Schares G, Vrhovec MG, Pantchev N, Herrmann DC, Conraths FJ. Occurrence of *Toxoplasma gondii* and Hammondia hammondi oocysts in the faeces of cats from Germany and other European countries. Vet Parasitol. 2008;152(1–2):34–45. doi: 10.1016/j.vetpar.2007.12.004 18226453

[30] Khan A, Dubey JP, Su C, Ajioka JW, Rosenthal BM, Sibley LD. Genetic analyses of atypical *Toxoplasma gondii* strains reveal a fourth clonal lineage in North America. Int J Parasitol. 2011;41(6):645–55. doi: 10.1016/j.ijpara.2011.01.005 21320505 PMC3081397

[31] Mercier A, Ajzenberg D, Devillard S, Demar MP, de Thoisy B, Bonnabau H, et al. Human impact on genetic diversity of *Toxoplasma gondii*: example of the anthropized environment from French Guiana. Infect Genet Evol. 2011;11(6):1378–87. doi: 10.1016/j.meegid.2011.05.003 21600306

[32] Jiang T, Shwab EK, Martin RM, Gerhold RW, Rosenthal BM, Dubey JP, et al. A partition of *Toxoplasma gondii* genotypes across spatial gradients and among host species, and decreased parasite diversity towards areas of human settlement in North America. Int J Parasitol. 2018;48(8):611–9. doi: 10.1016/j.ijpara.2018.01.008 29577892

[33] Hammond DS. Tropical forests of the Guiana Shield: ancient forests in a modern world. CABI. 2005. doi: 10.1079/9780851995366.0000

[34] de Thoisy B, Fayad I, Clément L, Barrioz S, Poirier E, Gond V. Predators, prey and habitat structure: can key conservation areas and early signs of population collapse be detected in neotropical forests? PLoS One. 2016;11(11):e0165362. doi: 10.1371/journal.pone.0165362 27828993 PMC5102489

[35] Kelly M, Morin D, Lopez-Gonzalez CA. Lynx rufus. The IUCN Red List of Threatened Species. 2016: e. T12521A50655874. 2019.

[36] Dumètre A, Ajzenberg D, Rozette L, Mercier A, Dardé M-L. *Toxoplasma gondii* infection in sheep from Haute-Vienne, France: seroprevalence and isolate genotyping by microsatellite analysis. Vet Parasitol. 2006;142(3–4):376–9. doi: 10.1016/j.vetpar.2006.07.005 16919879

[37] Aubert D, Ajzenberg D, Richomme C, Gilot-Fromont E, Terrier ME, de Gevigney C, et al. Molecular and biological characteristics of *Toxoplasma gondii* isolates from wildlife in France. Vet Parasitol. 2010;171(3–4):346–9. doi: 10.1016/j.vetpar.2010.03.033 20417034

[38] Ajzenberg D, Collinet F, Aubert D, Villena I, Dardé M-L, French ToxoBs network group, et al. The rural-urban effect on spatial genetic structure of type II *Toxoplasma gondii* strains involved in human congenital toxoplasmosis, France, 2002–2009. Infect Genet Evol. 2015;36:511–6. doi: 10.1016/j.meegid.2015.08.025 26305624

[39] Chaichan P, Mercier A, Galal L, Mahittikorn A, Ariey F, Morand S, et al. Geographical distribution of *Toxoplasma gondii* genotypes in Asia: a link with neighboring continents. Infect Genet Evol. 2017;53:227–38. doi: 10.1016/j.meegid.2017.06.002 28583867

[40] Vandel J-M, Stahl P. Distribution trend of the Eurasian lynx Lynx lynx populations in France. 2005.

[41] Lau MW-N, Fellowes JR, Chan BPL. Carnivores (Mammalia: Carnivora) in South China: a status review with notes on the commercial trade. Mammal Review. 2010;40(4):247–92. doi: 10.1111/j.1365-2907.2010.00163.x

[42] Bolais PF, Galal L, Cronemberger C, Pereira F de A, Barbosa A da S, Dib LV, et al. *Toxoplasma gondii* in the faeces of wild felids from the Atlantic forest, Brazil. Mem Inst Oswaldo Cruz. 2022;117:e210302. doi: 10.1590/0074-02760210302 35766781 PMC9241165

[43] Bullock EL, Woodcock CE, Souza C Jr, Olofsson P. Satellite-based estimates reveal widespread forest degradation in the Amazon. Glob Chang Biol. 2020;26(5):2956–69. doi: 10.1111/gcb.15029 32022338

[44] Mikissa JB, Pambou FK, Ngoyi EB. Biodiversity in Gabon: An overview. Global Biodiversity: Volume 3: Selected Countries in Africa. 2018; 63.

[45] Galal L, Sarr A, Cuny T, Brouat C, Coulibaly F, Sembène M, et al. The introduction of new hosts with human trade shapes the extant distribution of *Toxoplasma gondii* lineages. PLoS Negl Trop Dis. 2019;13(7):e0007435. doi: 10.1371/journal.pntd.0007435 31295245 PMC6622481

[46] Račka K, Bártová E, Hamidović A, Plault N, Kočišová A, Camacho G, et al. First detection of *Toxoplasma gondii* Africa 4 lineage in a population of carnivores from South Africa. Front Cell Infect Microbiol. 2024;14:1274577. doi: 10.3389/fcimb.2024.1274577 38352059 PMC10861644

[47] Su C, Shwab EK, Zhou P, Zhu XQ, Dubey JP. Moving towards an integrated approach to molecular detection and identification of *Toxoplasma gondii*. Parasitology. 2010;137(1):1–11. doi: 10.1017/S0031182009991065 19765337

[48] Gebremedhin EZ, Agonafir A, Tessema TS, Tilahun G, Medhin G, Vitale M, et al. Seroepidemiological study of ovine toxoplasmosis in East and West Shewa Zones of Oromia regional state, Central Ethiopia. BMC Vet Res. 2013;9:117. doi: 10.1186/1746-6148-9-117 23768427 PMC3685547

[49] Kaczensky P, Chapron G, von Arx M, Huber D, Andrén H, Linnell J. Status, management and distribution of large carnivores–bear, lynx, wolf & wolverine–in Europe. Document prepared with the assistance of Istituto di Ecologia Applicata and with the contributions of the IUCN/SSC Large Carnivore Initiative for Europe under contract N 070307. 2012.

[50] Scherrer P, Ryser-Degiorgis M-P, Marti IA, Borel S, Frey CF, Mueller N, et al. Exploring the epidemiological role of the Eurasian lynx (Lynx lynx) in the life cycle of *Toxoplasma gondii*. Int J Parasitol Parasites Wildl. 2023;21:1–10. doi: 10.1016/j.ijppaw.2023.03.005 37032843 PMC10074407

[51] Bertranpetit E, Jombart T, Paradis E, Pena H, Dubey J, Su C, et al. Phylogeography of *Toxoplasma gondii* points to a South American origin. Infect Genet Evol. 2017;48:150–5. doi: 10.1016/j.meegid.2016.12.020 28028000

[52] Zhang WQ, Zhang MH. Complete mitochondrial genomes reveal phylogeny relationship and evolutionary history of the family Felidae. Genet Mol Res. 2013;12(3):3256–62. doi: 10.4238/2013.September.3.1 24065666

[53] Aramini JJ, Stephen C, Dubey JP. *Toxoplasma gondii* in vancouver Island cougars (Felis concolor vancouverensis): serology and oocyst shedding. J Parasitol. 1998;84(2):438–40. 9576522

[54] Miller MA, Newberry CA, Sinnott DM, Batac FI, Greenwald K, Reed A, et al. Newly detected, virulent *Toxoplasma gondii* COUG strain causing fatal steatitis and toxoplasmosis in southern sea otters (Enhydra lutris nereis). Front Mar Sci. 2023;10. doi: 10.3389/fmars.2023.1116899

[55] Dubey JP, Alvarado-Esquivel C, Herrera-Valenzuela VH, Ortiz-Diaz JJ, Oliveira S, Verma SK, et al. A new atypical genotype mouse virulent strain of *Toxoplasma gondii* isolated from the heart of a wild caught puma (Felis concolor) from Durango, Mexico. Vet Parasitol. 2013;197(3–4):674–7. doi: 10.1016/j.vetpar.2013.06.005 23849518

[56] Su C, Khan A, Zhou P, Majumdar D, Ajzenberg D, Dardé M-L, et al. Globally diverse *Toxoplasma gondii* isolates comprise six major clades originating from a small number of distinct ancestral lineages. Proc Natl Acad Sci U S A. 2012;109(15):5844–9. doi: 10.1073/pnas.1203190109 22431627 PMC3326454

[57] Bowie WR, King AS, Werker DH, Isaac-Renton JL, Bell A, Eng SB, et al. Outbreak of toxoplasmosis associated with municipal drinking water. the BC toxoplasma investigation team. Lancet. 1997;350(9072):173–7. doi: 10.1016/s0140-6736(96)11105-3 9250185

[58] Carme B, Bissuel F, Ajzenberg D, Bouyne R, Aznar C, Demar M, et al. Severe acquired toxoplasmosis in immunocompetent adult patients in French Guiana. J Clin Microbiol. 2002;40(11):4037–44. doi: 10.1128/JCM.40.11.4037-4044.2002 12409371 PMC139686

[59] Demar M, Ajzenberg D, Maubon D, Djossou F, Panchoe D, Punwasi W, et al. Fatal outbreak of human toxoplasmosis along the Maroni river: epidemiological, clinical, and parasitological aspects. Clin Infect Dis. 2007;45(7):e88-95. doi: 10.1086/521246 17806043

[60] Schumacher AC, Elbadawi LI, DeSalvo T, Straily A, Ajzenberg D, Letzer D, et al. Toxoplasmosis outbreak associated with *Toxoplasma gondii*-contaminated venison-high attack rate, unusual clinical presentation, and atypical genotype. Clin Infect Dis. 2021;72(9):1557–65. doi: 10.1093/cid/ciaa285 32412062 PMC9248295

