# Supplementary material for: Toxoplasma gondii from Gabonese forest, Central Africa: First report of an African wild strain
Source: PLoS Negl Trop Dis. 2025 Jan 6;19(1):e0012214. doi: 10.1371/journal.pntd.0012214 (PMC13480050; doi:10.1371/journal.pntd.0012214)
Supplement: Supplementary file 2 [file pntd.0012214.s001.docx]

a. PORTUGAL10 (type I)

| Reference genomes | Number of reads |
| --- | --- |
| GT1 | 85088 |
| ME49 | 2436 |
| VEG | 1736 |
| MAS | 861 |
| RUB | 2052 |
| FOU | 1972 |
| TgCtBr9 | 628 |
| CAST | 2122 |
| TgCtBr5 | 551 |
| P89 | 878 |
| VAND | 1796 |
| COUG | 1157 |
| ARI | 711 |
| TgCtPRC2 | 1578 |
| GAB2-2007-GAL-DOM2 | 1328 |
| TgCtCo5 | 968 |

b. FRANCE01 (type II)

| Reference genomes | Number of reads |
| --- | --- |
| GT1 | 1887 |
| ME49 | 154567 |
| VEG | 5848 |
| MAS | 1543 |
| RUB | 1599 |
| FOU | 549 |
| TgCtBr9 | 363 |
| CAST | 946 |
| TgCtBr5 | 729 |
| P89 | 1152 |
| VAND | 2037 |
| COUG | 4211 |
| ARI | 6335 |
| TgCtPRC2 | 5001 |
| GAB2-2007-GAL-DOM2 | 2779 |
| TgCtCo5 | 1607 |

c. PORTUGAL09 (type III)

| Reference genomes | Number of reads |
| --- | --- |
| GT1 | 2901 |
| ME49 | 10097 |
| VEG | 164397 |
| MAS | 2142 |
| RUB | 3700 |
| FOU | 1146 |
| TgCtBr9 | 1505 |
| CAST | 1961 |
| TgCtBr5 | 1571 |
| P89 | 3246 |
| VAND | 3067 |
| COUG | 3143 |
| ARI | 2739 |
| TgCtPRC2 | 3125 |
| GAB2-2007-GAL-DOM2 | 5277 |
| TgCtCo5 | 1680 |

d. BENIN01 (Africa 1)

| Reference genomes | Number of reads |
| --- | --- |
| GT1 | 8600 |
| ME49 | 1819 |
| VEG | 1902 |
| MAS | 936 |
| RUB | 2090 |
| FOU | 19203 |
| TgCtBr9 | 1407 |
| CAST | 2422 |
| TgCtBr5 | 591 |
| P89 | 936 |
| VAND | 1830 |
| COUG | 1199 |
| ARI | 712 |
| TgCtPRC2 | 1574 |
| GAB2-2007-GAL-DOM2 | 3538 |
| TgCtCo5 | 1772 |

e. SENEGAL04 (Africa 4)

| Reference genomes | Number of reads |
| --- | --- |
| GT1 | 3220 |
| ME49 | 20611 |
| VEG | 2252 |
| MAS | 15726 |
| RUB | 11332 |
| FOU | 1188 |
| TgCtBr9 | 561 |
| CAST | 4621 |
| TgCtBr5 | 1428 |
| P89 | 3830 |
| VAND | 11267 |
| COUG | 19169 |
| ARI | 5066 |
| TgCtPRC2 | 279282 |
| GAB2-2007-GAL-DOM2 | 965 |
| TgCtCo5 | 5483 |

f. FRENCHGUIANA08 (Amazonian)

| Reference genomes | Number of reads |
| --- | --- |
| GT1 | 14582 |
| ME49 | 4883 |
| VEG | 12939 |
| MAS | 21259 |
| RUB | 72882 |
| FOU | 3803 |
| TgCtBr9 | 1402 |
| CAST | 13807 |
| TgCtBr5 | 5882 |
| P89 | 21895 |
| VAND | 67685 |
| COUG | 17520 |
| ARI | 5141 |
| TgCtPRC2 | 11559 |
| GAB2-2007-GAL-DOM2 | 3605 |
| TgCtCo5 | 21104 |

g. FRENCHGUIANA10 (Pan-American)

| Reference genomes | Number of reads |
| --- | --- |
| GT1 | 3149 |
| ME49 | 7988 |
| VEG | 2646 |
| MAS | 5146 |
| RUB | 7212 |
| FOU | 811 |
| TgCtBr9 | 541 |
| CAST | 2979 |
| TgCtBr5 | 1087 |
| P89 | 3119 |
| VAND | 7011 |
| COUG | 251780 |
| ARI | 16067 |
| TgCtPRC2 | 9245 |
| GAB2-2007-GAL-DOM2 | 764 |
| TgCtCo5 | 4236 |

h. MARTINIQUE01 (hybrid of Pan-American, type II and type III)

| Reference genomes | Number of reads |
| --- | --- |
| GT1 | 3256 |
| ME49 | 16166 |
| VEG | 20494 |
| MAS | 2332 |
| RUB | 3738 |
| FOU | 568 |
| TgCtBr9 | 570 |
| CAST | 1628 |
| TgCtBr5 | 814 |
| P89 | 2020 |
| VAND | 3094 |
| COUG | 190785 |
| ARI | 5593 |
| TgCtPRC2 | 4361 |
| GAB2-2007-GAL-DOM2 | 2550 |
| TgCtCo5 | 1942 |
