# Supplementary material for: Toxoplasma gondii from Gabonese forest, Central Africa: First report of an African wild strain
Source: PLoS Negl Trop Dis. 2025 Jan 6;19(1):e0012214. doi: 10.1371/journal.pntd.0012214 (PMC13480050; doi:10.1371/journal.pntd.0012214)
Supplement: Supplementary file 3 [file pntd.0012214.s007.docx]

Dear Dr. Fritz,
 
Thank you very much for submitting your manuscript "Toxoplasma gondii from Gabonese forest, Central Africa: first report of an African wild population" for consideration at PLOS Neglected Tropical Diseases. As with all papers reviewed by the journal, your manuscript was reviewed by members of the editorial board and by several independent reviewers. In light of the reviews (below this email), we would like to invite the resubmission of a significantly-revised version that takes into account the reviewers' comments.  


 
We cannot make any decision about publication until we have seen the revised manuscript and your response to the reviewers' comments. Your revised manuscript is also likely to be sent to reviewers for further evaluation.
 
When you are ready to resubmit, please upload the following:

[1] A letter containing a detailed list of your responses to the review comments and a description of the changes you have made in the manuscript. Please note while forming your response, if your article is accepted, you may have the opportunity to make the peer review history publicly available. The record will include editor decision letters (with reviews) and your responses to reviewer comments. If eligible, we will contact you to opt in or out.

[2] Two versions of the revised manuscript: one with either highlights or tracked changes denoting where the text has been changed; the other a clean version (uploaded as the manuscript file).
Important additional instructions are given below your reviewer comments.

Please prepare and submit your revised manuscript within 60 days. If you anticipate any delay, please let us know the expected resubmission date by replying to this email. Please note that revised manuscripts received after the 60-day due date may require evaluation and peer review similar to newly submitted manuscripts.
 
Thank you again for your submission. We hope that our editorial process has been constructive so far, and we welcome your feedback at any time. Please don't hesitate to contact us if you have any questions or comments.
 
Sincerely,

Claudia Ida Brodskyn
Section Editor
PLOS Neglected Tropical Diseases

Claudia Brodskyn
Section Editor
PLOS Neglected Tropical Diseases

***********************


Reviewer's Responses to Questions

AUTHOR’S RESPONSE:

Dear Editor,

Thank you for the attention you have dedicated to our manuscript. We greatly appreciate the valuable comments and suggestions from the reviewers and yourself, which have significantly helped improve the manuscript.

Key Review Criteria Required for Acceptance?
As you describe the new analyses required for acceptance, please consider the following:

Methods
-Are the objectives of the study clearly articulated with a clear testable hypothesis stated?
-Is the study design appropriate to address the stated objectives?
-Is the population clearly described and appropriate for the hypothesis being tested?
-Is the sample size sufficient to ensure adequate power to address the hypothesis being tested?
-Were correct statistical analysis used to support conclusions?
-Are there concerns about ethical or regulatory requirements being met?

Reviewer #1: Whit Editor

Reviewer #2: Minor re-organisation and additional information is needed in the Methodology section and specifics have been highlighted directly in the manuscript.

AUTHOR’S RESPONSE: We thank the reviewer for these positive remarks.

Reviewer #3: (No Response)

--------------------

Results
-Does the analysis presented match the analysis plan?
-Are the results clearly and completely presented?
-Are the figures (Tables, Images) of sufficient quality for clarity?

Reviewer #1: Whit Editor

Reviewer #2: The results are clearly presented including the Figures and Tables.

AUTHOR’S RESPONSE: We thank the reviewer for this positive remark.

Reviewer #3: 1. Please add percent prevalence, and how it tracked among the 7 wild mammal species sampled. It would be helpful to add common names, and whether the species are carnivores, herbivores, or omnivores in a table format.

AUTHOR’S RESPONSE: We thank the reviewer for this suggestion. We added this data in the new version of the manuscript. We also performed comparisons of prevalence levels between species.

2. In the same table, it would be helpful to list the qPCR result, to gauge the success of the 15 locus typing schematic.

AUTHOR’S RESPONSE: We thank the reviewer for this suggestion. We added this data in the new version of the manuscript, but in a separate table in supplementary data (S1 table).

3. Publications studying T. gondii genotypes infecting wildlife samples have demonstrated that mixed strain T. gondii infections can occur, sometimes at high frequency, in the various target tissues examined herein – heterozygosity can dramatically impact consensus calling for SNPs and reak havoc on INDEL analyses, producing artefacts. What controls were run to obviate this concern.

AUTHOR’S RESPONSE: It is true that mixed infections can occur; however, in this instance, we would expect to observe double peaks for one microsatellite marker, which was not the case here.

4. Table 1 – is it possible to put a line that separates the two samples studied herein, from the 9 control samples that were added for comparative purposes.

AUTHOR’S RESPONSE: We thank the reviewer for this suggestion. We modified the table in the new version of the manuscript.

5. Fig 2 – please add the location of the 16 reference genome assemblies to the AFC graphic

AUTHOR'S RESPONSE: We thank the reviewer for this suggestion. We attempted to incorporate this information into the AFC graph; however, the dense clustering of data points in several areas of the graph rendered it impractical.
--------------------

Conclusions
-Are the conclusions supported by the data presented?
-Are the limitations of analysis clearly described?
-Do the authors discuss how these data can be helpful to advance our understanding of the topic under study?
-Is public health relevance addressed?

Reviewer #1: Whit Editor

Reviewer #2: Conclusions are well supported by the data and public relevance of the results and public health relevance is emphasized.

AUTHOR’S RESPONSE: We thank the reviewer for this positive remark.

Reviewer #3: The conclusions are not supported by the data presented. Only one sample was studied at both multi-locus resolution, and at WGS, not a population.

**AUTHOR’S RESPONSE:** We thank the reviewer for this comment. In the revised version of the manuscript, as suggested by the reviewer, we have added additional evidence supporting the existence of an undescribed Toxoplasma gondii population in the forests of Gabon: 'Gabon-87_2019-Cephalophus-sp (ID: 87), a heart sample from a Cephalophus sp. from Mekouma (12/15), and Gabon-21_2019-Cephalophus-callipygus (ID: 21), a heart sample from a Cephalophus callipygus from Zoula (1/15). Both samples displayed a novel allele (228 at M102), which has not been previously observed in any of the published MLGs, suggesting they may belong to the same population. This allele was highly divergent from all other alleles reported for this MS marker, which shows allele sizes ranging from 164 to 196, except for TgSpEt19, a strain from a sheep in Ethiopia, which displayed a fragment of 218 at this marker.' Additionally, the genetic proximity between Gabon-87_2019-Cephalophus-sp and TgSpEt19, along with their divergence from the rest of global diversity, suggests that this T. gondii population has a wide geographical range across Africa. Overall, we can infer that the isolation of a single individual indicates the presence of a distinct population.

Further, insufficient depth of coverage was obtained to resolve the genome with high confidence.

**AUTHOR’S RESPONSE:** In the revised version of the manuscript, we updated our mapping approach by using FastQ Screen software, configured to utilize the bwa-mem2 alignment tool. FastQ Screen provides information on the proportion of reads that map specifically (with emphasis on the word 'specifically') to each of the selected reference genomes. Crucially, we validated the robustness of this approach by testing it on eight *T. gondii* genomes with known phylogenetic classification and genomic ancestry composition (S1 Fig.). We added a sentence emphasizing the limitations of this approach in describing phylogenetic positioning or ancestry characterization. However, the whole-genome approach developed in the present study provides valuable insights into the relative genomic relatedness between new *T. gondii* genomes and global *T. gondii* haplogroups in a context of low read count, as we expect in most organ samples contaminated with *T. gondii*. Overall, we believe that, of course, in-depth genomic analyses require viable isolates, but that efforts also should be deployed in exploiting DNA directly extracted from clinical/animal/environmental in genomics.

Did the authors map reads to the 15 locus typing markers - and did the WGS analysis support the allele designation resolved by PCR?

**AUTHOR’S RESPONSE: This type of analysis is unfortunately not possible in a context of low read count** as we expect in most organ samples contaminated with *T. gondii*.

Was there evidence in either the PCR or WGS analysis, for mixed strain superinfection of the tissue?

**AUTHOR’S RESPONSE:** This is a very interesting point. While mixed infections can indeed occur, in this case, we would expect to see double peaks for at least one microsatellite marker, which was not observed.

To control for any potential artifact in the SNP calling pipelines to resolve the allele present. Much of the discussion centers on the Gabon strain being genetically related to the COUG isolate - but for the reasons above, this is circumspect, and not sufficiently supported, and the present study, in my opinion, does not provide evidence for the existence of a sylvatic cycle of T. gondii in Africa [lines 312-314].

**AUTHOR’S RESPONSE:** The genetic proximity between Gabon-87_2019-Cephalophus-sp and TgSpEt19, along with their divergence from the rest of global diversity, suggests that this T. gondii population has a wide geographical range across Africa.

Importantly, they do not provide evidence that an African wild T. gondii strain
has emerged with a potential to cause severe toxoplasmosis among immunocompetent individuals - which has been demonstrated to occur in South and North America, but not herein.

**AUTHOR’S RESPONSE: We totally agree with the reviewer on this point. This is a hypothesis extrapolated from what is observed in South and North America. That is why we are concluding our study with: “**The involvement of African wild *T. gondii* strains in the incidence of severe toxoplasmosis among immunocompetent individuals requires further investigation.”

--------------------

Editorial and Data Presentation Modifications?
Use this section for editorial suggestions as well as relatively minor modifications of existing data that would enhance clarity. If the only modifications needed are minor and/or editorial, you may wish to recommend “Minor Revision” or “Accept”.
Reviewer #1: Whit Editor

Reviewer #2: I have recommended minor re-organisation of information to enhance clarity, and this is highlighted directly on the manuscript specifically in the methodology section.

AUTHOR’S RESPONSE: We thank the reviewer for these positive remarks.

Reviewer #3: (No Response)

--------------------

 Summary and General Comments
Use this section to provide overall comments, discuss strengths/weaknesses of the study, novelty, significance, general execution and scholarship. You may also include additional comments for the author, including concerns about dual publication, research ethics, or publication ethics. If requesting major revision, please articulate the new experiments that are needed.

Reviewer #1: Whit Editor

Reviewer #2: The authors report on a study titled “Toxoplasma gondii from Gabonese forest, Central Africa: first report of an African wild
population”. I have carefully gone through the contents of the manuscript and the authors have rightfully justified the purpose of the study. The methodologies followed are sound and I have indicated my specific comments to enhance clarity directly on the manuscript for easy of revisions.

AUTHOR’S RESPONSE: We thank the reviewer for this positive remark.

The study villages are only quantified without support of information on the geographical location of these in Gabon. My suggestion is for the authors to consider adding a map from their already published paper in this journal (Pierre Becquart et. al., 2024) which used the same study sites. This will assist the readership understanding without having to look for the publication with this information.

AUTHOR’S RESPONSE: We thank the reviewer for this suggestion. We added a map in the new version of the manuscript.

Reviewer #3: Toxoplasma gondii from Gabonese forest, Central Africa: first report of an African wild population

In this paper, 148 animals from at least seven species of wild mammals in Gabon Africa (collected as part of a viral screening program) were tested for the presence of Toxoplasma gondii (T. gondii) DNA by qPCR. T. gondii DNA was found in 15 animals from four different species that were collected from six villages, a prevalence of ~10%. The authors attempted to genotype the PCR positive samples using 15 typing markers that included a combination of 8 genotyping markers of limited variation and 7 fingerprinting microsatellite markers that are more varied across T. gondii lineages. Only one sample had sufficient DNA to yield a multi-locus genotype (at 12 of 15 markers). Two markers possessed new alleles, but it was not clear how divergent the new alleles were – whether they were different by a single SNP, an INDEL, or they were something more divergent.

AUTHOR’S RESPONSE: This is an interesting point that further highlights the divergent genotype of the new *Toxoplasma gondii* strains identified in the present study. This aspect has been addressed in the revised version of the manuscript.

In the NJ tree, the Gabon-87 sample was distinct, and resolved on a single branch away from the majority of other MLGs, and it resolved closely with another isolate from a Sheep in Ethiopia. Is it known whether the animal infected with Gabon-87 was sick, and impacted by toxoplasmosis?

AUTHOR’S RESPONSE: No signs of disease (e.g., lesions or abnormal appearance) were detected on the animal’s organs. This relevant point was added to the revised version of the manuscript.

Likewise for the Sheep from Ethiopia, as the paper discusses the relevance of the emergence of highly pathogenic strains from wildlife reservoirs. Using their published database of 1068 MLGs, a Factorial correspondence analysis (AFC) supported their divergent designation as both the sample from Ethiopia (not studied herein) and the Gabon-87 sample were distinct from the majority of other samples (Fig. 2) at the 12 loci examined. It would have been informative to have labeled Fig. 2 with the position of FOU (Africa 1), COUG, VEG and TgCtBr5, for example, the 4 reference genomes that had the most reads map from the Gabon-87 unenriched WGS sequencing effort.

AUTHOR'S RESPONSE: We agree with the reviewer and we attempted to incorporate this information into the AFC graph; however, the dense clustering of data points in several areas of the graph rendered it impractical.

In an attempt to gather more T. gondii sequence from the infected tissue sample, a total of 1,371,024,686 paired-end illumina reads were obtained from the infected heart sample, of which 282,006 (representing 0.02% of trimmed reads) were T. gondii. This would represent about 84.6Mb of sequence assuming 300bp of high quality sequence was obtained for each paired read, or marginally 1X genome coverage. Not sure how they calculated an average depth of genome coverage at 3.5X [lines 217-218]?

AUTHOR’S RESPONSE: The calculation method we used did not account for uncovered positions (positions with zero reads), which was an error. In the revised version of the manuscript, we removed this section, acknowledging that the estimated average depth is highly dependent on the selected reference genome.

In a novel attempt to resolve genetic relatedness, the Gabon-87 reads were mapped against the 16 T. gondii reference genome assemblies at various mapping stringencies, and the majority of reads mapped, in descending order, to COUG, FOU (Africa 1), TgCtBr5 and VEG. The authors concluded that the Gabon-87 sample was more related to COUG than to the major domestic T. gondii lineages found in Gabon and Africa (type II, Africa 1, Africa 3) [lines 298-300], which I find difficult to support. While ~200K reads did map to the COUG assembly at -γ 0.98 using smalt, ~100K reads mapped to the Africa 1 FOU assembly, ~80K reads to the TgCtBr5 assembly, and ~75K to the VEG (type III) assembly. Is this a novel recombinant? Did the mapped reads overlap for each assembly, or were they in separate parts of the genome? Clearly a significant number of reads are mapping to an Africa 1 isolate, which is a major domestic lineage circulating in Gabon.

AUTHOR’S RESPONSE: All the points highlighted by the reviewers are indeed relevant. In the revised version of the manuscript, we updated our mapping approach by using FastQ Screen software, configured to utilize the bwa-mem2 alignment tool. FastQ Screen provides information on the proportion of reads that map specifically (with emphasis on the word 'specifically') to each of the selected reference genomes. Crucially, we validated the robustness of this approach by testing it on eight *T. gondii* genomes with known phylogenetic classification and genomic ancestry composition (S1 Fig.). The results differ slightly, but the conclusions remain largely the same. We added a sentence emphasizing the limitations of this approach in describing phylogenetic positioning or ancestry characterization. Despite these limitations, the whole-genome approach developped in the present study provides valuable insights into the relative genomic relatedness between new *T. gondii* genomes and global *T. gondii* haplogroups in a context of low read count, as we expect in most organ samples contaminated with *T. gondii*. As emphasized above, we believe that efforts also should be deployed in exploiting DNA directly extracted from clinical/animal/environmental in genomics.

Hence, without enrichment, or an isolate, the authors can only infer the origin of the sample, and they only have data for one sample. However, the title suggests the existence of an African wild population of circulating T. gondii, but the analysis is for only one sample, so it is incomplete.

AUTHOR’S RESPONSE: We agree with the reviewer on for need of viable isolates to conduct in-depth genomic analyses on this new population. That is exactly what we hope for in the near future.

--------------------

PLOS authors have the option to publish the peer review history of their article (<a href=["https://journals.plos.org/plosntds/s/editorial-and-peer-review-process#loc-peer-review-history"](https://journals.plos.org/plosntds/s/editorial-and-peer-review-process#loc-peer-review-history) target="_blank">what does this mean?). If published, this will include your full peer review and any attached files

If you choose “no”, your identity will remain anonymous but your review may still be made public

<b>Do you want your identity to be public for this peer review?</b> For information about this choice, including consent withdrawal, please see our <a href=["https://www.plos.org/privacy-policy"](https://www.plos.org/privacy-policy) target="_blank">Privacy Policy</a>.

Reviewer #1: No

Reviewer #2: Yes: Samson Mukaratirwa

Reviewer #3: No
