# Supplementary material for: Toxoplasma gondii from Gabonese forest, Central Africa: First report of an African wild strain
Source: PLoS Negl Trop Dis. 2025 Jan 6;19(1):e0012214. doi: 10.1371/journal.pntd.0012214 (PMC13480050; doi:10.1371/journal.pntd.0012214)
Supplement: Supplementary file 4 [file pntd.0012214.s008.docx]

PNTD-D-24-00685R1

Toxoplasma gondii from Gabonese forest, Central Africa: first report of an African wild population

PLOS Neglected Tropical Diseases

Dear Dr. Fritz,

Thank you for submitting your manuscript to PLOS Neglected Tropical Diseases. After careful consideration, we feel that it has merit but does not fully meet PLOS Neglected Tropical Diseases's publication criteria as it currently stands. Therefore, we invite you to submit a revised version of the manuscript that addresses the points raised during the review process.

Please submit your revised manuscript within 30 days Dec 12 2024 11:59PM. If you will need more time than this to complete your revisions, please reply to this message or contact the journal office at plosntds@plos.org. When you're ready to submit your revision, log on to https://www.editorialmanager.com/pntd/ and select the 'Submissions Needing Revision' folder to locate your manuscript file.

Please include the following items when submitting your revised manuscript:

* A rebuttal letter that responds to each point raised by the editor and reviewer(s). You should upload this letter as a separate file labeled 'Response to Reviewers'. This file does not need to include responses to any formatting updates and technical items listed in the 'Journal Requirements' section below.

* A marked-up copy of your manuscript that highlights changes made to the original version. You should upload this as a separate file labeled 'Revised Manuscript with Track Changes'.

* An unmarked version of your revised paper without tracked changes. You should upload this as a separate file labeled 'Manuscript'.

If you would like to make changes to your financial disclosure, competing interests statement, or data availability statement, please make these updates within the submission form at the time of resubmission. Guidelines for resubmitting your figure files are available below the reviewer comments at the end of this letter.

We look forward to receiving your revised manuscript.

Kind regards,

Claudia Ida Brodskyn

Section Editor

PLOS Neglected Tropical Diseases

Claudia Brodskyn

Section Editor

PLOS Neglected Tropical Diseases

Shaden Kamhawi
co-Editor-in-Chief
PLOS Neglected Tropical Diseases
orcid.org/0000-0003-4304-636XX

Paul Brindley
co-Editor-in-Chief
PLOS Neglected Tropical Diseases
orcid.org/0000-0003-1765-0002

**Journal Requirements:**

**Additional Editor Comments (if provided):**

[Note: HTML markup is below. Please do not edit.]

**Reviewers' comments:**

Reviewer's Responses to Questions

**Key Review Criteria Required for Acceptance?**


As you describe the new analyses required for acceptance, please consider the following:


**Methods**


-Are the objectives of the study clearly articulated with a clear testable hypothesis stated?

-Is the study design appropriate to address the stated objectives?

-Is the population clearly described and appropriate for the hypothesis being tested?

-Is the sample size sufficient to ensure adequate power to address the hypothesis being tested?

-Were correct statistical analysis used to support conclusions?

-Are there concerns about ethical or regulatory requirements being met?

Reviewer #4: This study is clear in aims and the results provide new clue information on Toxoplasma diversity

Reviewer #5: Overall the methods are sounds, objectives are clearly stated and addressed. Small number (N=2) of samples were successfully characterized, however this weakness is addressed by the authors.

Reviewer #6: -Are the objectives of the study clearly articulated with a clear testable hypothesis stated?
YES

-Is the study design appropriate to address the stated objectives?
YES

-Is the population clearly described and appropriate for the hypothesis being tested?
YES

-Is the sample size sufficient to ensure adequate power to address the hypothesis being tested?
Detaileed data on a single isolate only appears not enough to conclude on a wild population.

AUTHOR’S RESPONSE: We thank the reviewer for this remark, and we have replaced the term “population” by “strain” throughout the manuscript.

-Were correct statistical analysis used to support conclusions?
Not applicable.

-Are there concerns about ethical or regulatory requirements being met?
NO

**Results**


-Does the analysis presented match the analysis plan?

-Are the results clearly and completely presented?

-Are the figures (Tables, Images) of sufficient quality for clarity?

Reviewer #4: The information was obtained with methods that are adequate for the purpose of the study and figures are informative

Reviewer #5: The analysis is sound and most of the figures are clearly provided. One figure lacks axes labels (noted in comments).

AUTHOR’S RESPONSE: We thank the reviewer for her positive remark. Axes labels have been added in the new version of the manuscript.

Reviewer #6: -Does the analysis presented match the analysis plan?
YES

-Are the results clearly and completely presented?
YES

-Are the figures (Tables, Images) of sufficient quality for clarity?
Figure 4 and Supplementary Figures need axes descriptions. Furthermore I am a bit puzzled - There is a Fig4 in the text and a Fig4ab after the text, i.e. there are two different Fig. 4. ???

AUTHOR’S RESPONSE: We thank the reviewer for this remark. Axes labels have been added in the new version of the manuscript. There was a Fig4ab in an anterior version of the manuscript; but it should still be appearing in the tracked-changes version of the manuscript only. There is only a single Fig. 4 now, without subdivisions.

**Conclusions**


-Are the conclusions supported by the data presented?

-Are the limitations of analysis clearly described?

-Do the authors discuss how these data can be helpful to advance our understanding of the topic under study?

-Is public health relevance addressed?

Reviewer #4: The discussion is well supported

Reviewer #5: The limitation of sample size is acknowledged by the authors. A major gap in knowledge on the global epidemiology of Toxoplasma is lack of studies in Africa, which this paper strives to address. The authors note that additional studies will be needed for more clarity on population genetics of the parasite in this continent, as well as to understand whether certain strains are associated with more virulent disease in this region.

Reviewer #6: -Are the conclusions supported by the data presented?

Line 1 -2 "population" goes too far, to my opinion. I suggest the following title or similar: "A unique Toxoplasma gondii genotype from Gabonese forest suggests the excistence of an African wild toxoplasma population "

Line 305: It is impossible for me to accept the use of "T. gondii population" if only a single representative (i.e. a single isolate) has been identified yet. I suggest to rephrase.

AUTHOR’S RESPONSE: As emphasized above, we refined the manuscript accordingly, , with the substitution of the term "population" by “strain”.

Couldn't this single isolate be the result of sexual recombination of representatives of true T. gondii populations?

AUTHOR’S RESPONSE: If the single isolate resulted from sexual recombination between representatives of the more common T. gondii lineages (assuming this is what the reviewer means), its microsatellite allelic profile would likely be a mixture of the microsatellite alleles associated with these lineages (Africa 1, Africa 3, and Type III in Gabon). Additionally, regarding genomic analysis, we would expect to observe more reads specifically mapping to the reference genomes representing these three lineages (FOU for Africa 1, GAB2-2007-GAL-DOM2 for Africa 3, and VEG for Type III).

-Are the limitations of analysis clearly described?
YES

-Do the authors discuss how these data can be helpful to advance our understanding of the topic under study?
YES

-Is public health relevance addressed?
YES

**Editorial and Data Presentation Modifications?**


Use this section for editorial suggestions as well as relatively minor modifications of existing data that would enhance clarity. If the only modifications needed are minor and/or editorial, you may wish to recommend “Minor Revision” or “Accept”.

Reviewer #4: No suggestions

Reviewer #5: See notes in the next section

Reviewer #6: Further suggestions:
Line 59: "may mark" istead of "mark"
Line 74: "may mark" insrtead of "mark"

AUTHOR’S RESPONSE: We changed these sentences to “This discovery marks the first description suggests the existence of a wild *T. gondii* population in Africa.” To highlights the lack of very strong evidence.

There is no definition of "Wild strain" given in the introduction. Please add. Some examples on "wild strains" or "wild polulations" might also improve introduction.

AUTHOR’S RESPONSE: We added a definition of wild strains in the introduction. We agree with the reviewer on the interest of providing examples of wild populations, but we wanted to avoid redundancy with the discussion section, in which this aspect is discussed in details.

Line 128: Explain abbreviation "qPCR" already here and not in Line 139.

AUTHOR’S RESPONSE: We thank the reviewer for this remark. This was corrected accordingly.

Line 161: You had no results for 3 of the typing marker regions M33, IV.1 and XI.1. How did you treat this missing information when generating n-j-dendrogram?

AUTHOR’S RESPONSE: The NJ tree and the APC were produced based on the analysis of the 12 MS markers that were successfully amplified in Gabon-87_2019-Cephalophus-sp . The three other markers were not considered in the analyses.

Line 182: Please intoduce the abbreviation for "haplogroup" here.
Line 184: Please use only the abbreviation "hg" here.
Line 185: Please replace "haplogroup" by "hg"

AUTHOR’S RESPONSE: This was corrected accordingly.

Line 219: Please write "...M102, a fingerprinting marker". This is important because new alleles in finger printing markers are not such a surprise.

AUTHOR’S RESPONSE: This was corrected accordingly.

Line 257: What is the meaning of "uniquely mapping"?

AUTHOR’S RESPONSE: It means exclusively. We changed “uniquely” for “exclusively” in the new version of the manuscript.

Did these about 70 reads map exclusively to TgCtPRC2 ? Why are there two versions of Figure 4?

AUTHOR’S RESPONSE: Yes, 72 reads mapped exclusively against TgCtPRC2. There was a Fig4ab in an anterior version of the manuscript; but it should still be appearing in the tracked-changes version of the manuscript only. There is only a single Fig. 4 now, without subdivisions.

**Summary and General Comments**


Use this section to provide overall comments, discuss strengths/weaknesses of the study, novelty, significance, general execution and scholarship. You may also include additional comments for the author, including concerns about dual publication, research ethics, or publication ethics. If requesting major revision, please articulate the new experiments that are needed.

Reviewer #4: The authors described the presence, by PCR of Toxoplasma in wild species in Gabon (Africa). They sequenced one new complete genome. The descriptions add valuable information. The manuscript was well written. I have made some minor remarks.

AUTHOR’S RESPONSE: We are very grateful to the reviewer for their positive feedback.

- Please include the percentage of positive results by species. The reader can calculate this, but it can ease the lecture if percentages are shown. Please think of the readers.

AUTHOR’S RESPONSE: We are not sure to understand, since the percentage of positive results by species is provided in S2 table and quoted in the text of the manuscript (line 217)?

- Why do authors not estimate the molecular clock? I think it is important if any estimates between the strains are assayed by calibrating with genes as done in Plasmodium: Science. 2010;329(5988):226-9. doi: 10.1126/science.1188954

AUTHOR’S RESPONSE: We have indeed estimated the molecular clock of *T. gondii* in a previous study (Galal, et al. A unique *Toxoplasma gondii* haplotype accompanied the global expansion of cats. Nature Communications. 2022;13: 5778). However, since the genome assembly of our Gabon strain could not be completed due to low coverage, dating genome divergence times was not possible. This aspect would be fascinating to explore but will likely require high-quality genomes.

- Please include the database where the complete genome sequence was submitted and the accesion number.

AUTHOR’S RESPONSE: The following sentence has been added to the results section “Gabon-87_2019-Cephalophus-sp sequencing data have been deposited in the European Nucleotide Archive (ENA) database under accession code ERR13964874.”

Reviewer #5: This is a well written and timely manuscript on circulating genomic strains of Toxoplasma gondii in Gabon, Africa – a region vastly understudied for this parasite. The authors describe application of both an established (MS) as well as advanced (WGS) approach to evaluate genetic diversity of T. gondii strains. The scarcity of samples that contained high enough levels of parasite DNA for characterization is a weakness that was noted by the authors, as is the need to further evaluate whether ‘wild’ strains in Africa are associated with more severe toxoplasmosis in humans and animals. I have provided a few comments and edits for the authors’ consideration. Overall, this paper provides an important advance to the field of Toxoplasma epidemiology, especially given lack of studies on this globally important pathogen from the continent of Africa.

AUTHOR’S RESPONSE: We are sincerely grateful to the reviewer for her positive feedback.

Specific comments:
L48 - Compared with other globally prevalent pathogens, and even related apicomplexans (e.g. Sarcocystis spp), T. gondii is genetically quite conserved and rather limited in diversity, though this perception may depend on the context; this is just a note, not necessarily requiring authors to edit

AUTHOR’S RESPONSE: We thank the reviewer for these insights. Regarding the comparison between *Sarcocystis* spp. and *T. gondii*, we can assume that this difference may stem from the more specialized host adaptations exhibited by *Sarcocystis* spp. compared to the broader host range of *T. gondii*.

L60 - Could edit here and elsewhere from ‘population’ to 'strain' to address concerns from other reviewers that the lack of genetic characterization on more than two samples (or one for WGS) does not justify the term 'population' in this context.

AUTHOR’S RESPONSE: We have refined the manuscript accordingly by replacing the term “population” by “strain”.

L168 - Describe what you mean by 'high' (e.g. what Ct value exactly)?

AUTHOR’S RESPONSE: We acknowledge that the term "high" was not particularly objective. Consequently, we revised the sentence (line 173) to: "As a complement to MS genotyping, whole genome sequencing (WGS) was performed on successfully genotyped DNA samples."

L198 - Were positive qPCR results from screening assay confirmed via sequencing? Since the 529 RE target, while often described as Toxo-specific, can lead to non-specific amplification as well.

AUTHOR’S RESPONSE: This is a relevant remark. We have added these sentences to the discussion to highlight this point (line 283): “Molecular prevalence among species was relatively low but challenging to compare due to the small sample sizes for several species and the inaccurate identification of approximately one-third of the animals. In addition, the molecular prevalence levels may have been slightly underestimated due to the possible weak cross-reactivity of the primers targeting the T. gondii 529 bp repeat region with Hammondia DNA, as previously reported by Schares et al. (2008).”

L215 - What is typically considered low enough Ct to yield positive MS typing? It might be interesting to present the Ct results for the positive samples (as sup data). Was the successfully typed samples (N=2) notably lower Ct than other samples for which MS typing was not successful?

AUTHOR’S RESPONSE: This is an interesting (and somewhat challenging) question. It has been demonstrated that the same sample can yield different Ct values when amplified using different thermocyclers. For example, under varying conditions, Ct values differed by more than 3 units in some cases (Joeres et al., "A ring trial to harmonize *Toxoplasma gondii* microsatellite typing: comparative analysis of results and recommendations for optimization." European Journal of Clinical Microbiology & Infectious Diseases 42.7 (2023): 803-818). Consequently, relying on *T. gondii* cell copy number estimations appears to be more consistent and reliable than Ct values alone. In general, the presence of 100 *T. gondii* cell copies is sufficient to successfully amplify all 15 markers (Ajzenberg et al., "Genotyping of *Toxoplasma gondii* isolates with 15 microsatellite markers in a single multiplex PCR assay." J Clin Microbiol. 2010;48: 4641–4645. doi:10.1128/JCM.01152-10). We have added *T. gondii* cell copy number estimations in the new version of the manuscript.

L 215 – here and general question/comment – I’m curious as to whether the authors considered the nested PCR MLST approach commonly used in North America for genetically characterizing the qPCR-positive samples? the nested PCR approach may be more sensitive than MS typing for DNA characterization in tissues with low abundance of Tg DNA. The authors may wish to comment on the choice for genotyping approaches in the discussion, and other options that may allow for characterization of such samples (even if at lower resolution than MS typing).

AUTHOR’S RESPONSE: It is true that we have more experience in MS analysis, and that other genotyping techniques could provide complementary information or be useful in context of low *T. gondii* DNA concentration as it often occurs. We added this paragraph to the discussion to discuss the pros and cons of the different techniques (line 330): “Studies on the genetic diversity of *T. gondii* have revealed significant diversity that can only be effectively captured through multilocus genotyping methods, such as MS analysis, restriction fragment length polymorphism (RFLP), and multilocus sequence typing (MLST). Among these, MS genotyping offers higher resolution compared to RFLP and benefits from the availability of numerous MS genotypes published worldwide, making it more advantageous for comparative studies than MLST, despite the latter's higher sensitivity. While WGS offers an even more comprehensive approach, its implementation remains more challenging.”

Figures:
In-text Fig 4. Please add Y and X axes labels
Fig 4 with two panels on P. 25: Is this figure supposed to be in main paper? Above for Fig 4 within text I only see one panel for a similar figure
- And please add labels for axes. unclear if these are number of reads?

AUTHOR’S RESPONSE: Axes labels have been added in the new version of the manuscript. There was a Fig4ab in an anterior version of the manuscript; but it should still be appearing in the tracked-changes version of the manuscript only. There is only a single Fig. 4 now, without subdivisions.

Reviewer #6: This represents an interesting study providing an indication that there is more genotypic variation in Toxoplasma gondii in Africa than known yet.

AUTHOR’S RESPONSE: We are very grateful to the reviewer for their positive feedback.

PLOS authors have the option to publish the peer review history of their article ([what does this mean?](https://track.editorialmanager.com/CL0/https:%2F%2Fjournals.plos.org%2Fplosntds%2Fs%2Feditorial-and-peer-review-process%23loc-peer-review-history/1/010f01932036157d-ea9c8e19-c507-43d4-875a-0e4b334cf5e2-000000/pgjU8UJTOgvHUAN1OOa_Md0TCCQeNHpxilBdHIDLEAk=184)). If published, this will include your full peer review and any attached files.


If you choose “no”, your identity will remain anonymous but your review may still be made public.


**Do you want your identity to be public for this peer review?** For information about this choice, including consent withdrawal, please see our [Privacy Policy](https://track.editorialmanager.com/CL0/https:%2F%2Fwww.plos.org%2Fprivacy-policy/1/010f01932036157d-ea9c8e19-c507-43d4-875a-0e4b334cf5e2-000000/-isy0jR09m5h1d5UmWodM16shbHNNXrHIefMzRKH2Ig=184).

Reviewer #4: No

Reviewer #5: **Yes:** Karen Shapiro

Reviewer #6: No

[NOTE: If reviewer comments were submitted as an attachment file, they will be attached to this email and accessible via the submission site. Please log into your account, locate the manuscript record, and check for the action link "View Attachments". If this link does not appear, there are no attachment files.]

**Figure resubmission:**

While revising your submission, please upload your figure files to the Preflight Analysis and Conversion Engine (PACE) digital diagnostic tool, https://pacev2.apexcovantage.com/. PACE helps ensure that figures meet PLOS requirements. To use PACE, you must first register as a user. Registration is free. Then, login and navigate to the UPLOAD tab, where you will find detailed instructions on how to use the tool. If you encounter any issues or have any questions when using PACE, please email PLOS at figures@plos.org. Please note that Supporting Information files do not need this step. If there are other versions of figure files still present in your submission file inventory at resubmission, please replace them with the PACE-processed versions.

**Reproducibility:**

To enhance the reproducibility of your results, we recommend that authors of applicable studies deposit laboratory protocols in protocols.io, where a protocol can be assigned its own identifier (DOI) such that it can be cited independently in the future. Additionally, PLOS ONE offers an option to publish peer-reviewed clinical study protocols. Read more information on sharing protocols at https://plos.org/protocols?utm_medium=editorial-email&utm_source=authorletters&utm_campaign=protocols
