# Supplementary material for: Toxoplasma gondii from Gabonese forest, Central Africa: First report of an African wild strain
Source: PLoS Negl Trop Dis. 2025 Jan 6;19(1):e0012214. doi: 10.1371/journal.pntd.0012214 (PMC13480050; doi:10.1371/journal.pntd.0012214)
Supplement: Peer review history

# Peer review: Decision Letter 0

**DOI:** 10.1371/journal.pntd.0012214.r001

**Authors:** Claudia Ida Brodskyn

15 Jul 2024

Dear Dr. Fritz,

Thank you very much for submitting your manuscript "Toxoplasma gondii from Gabonese forest, Central Africa: first report of an African wild population" for consideration at PLOS Neglected Tropical Diseases. As with all papers reviewed by the journal, your manuscript was reviewed by members of the editorial board and by several independent reviewers. In light of the reviews (below this email), we would like to invite the resubmission of a significantly-revised version that takes into account the reviewers' comments. 

We cannot make any decision about publication until we have seen the revised manuscript and your response to the reviewers' comments. Your revised manuscript is also likely to be sent to reviewers for further evaluation.

When you are ready to resubmit, please upload the following:

[1] A letter containing a detailed list of your responses to the review comments and a description of the changes you have made in the manuscript. Please note while forming your response, if your article is accepted, you may have the opportunity to make the peer review history publicly available. The record will include editor decision letters (with reviews) and your responses to reviewer comments. If eligible, we will contact you to opt in or out.

[2] Two versions of the revised manuscript: one with either highlights or tracked changes denoting where the text has been changed; the other a clean version (uploaded as the manuscript file). 

Important additional instructions are given below your reviewer comments.

Please prepare and submit your revised manuscript within 60 days. If you anticipate any delay, please let us know the expected resubmission date by replying to this email. Please note that revised manuscripts received after the 60-day due date may require evaluation and peer review similar to newly submitted manuscripts.

Thank you again for your submission. We hope that our editorial process has been constructive so far, and we welcome your feedback at any time. Please don't hesitate to contact us if you have any questions or comments.

Sincerely,

Claudia Ida Brodskyn

Section Editor

PLOS Neglected Tropical Diseases

Claudia Brodskyn

Section Editor

PLOS Neglected Tropical Diseases

***********************

Reviewer's Responses to Questions

**Key Review Criteria Required for Acceptance?**

As you describe the new analyses required for acceptance, please consider the following:

**Methods**

-Are the objectives of the study clearly articulated with a clear testable hypothesis stated?

-Is the study design appropriate to address the stated objectives?

-Is the population clearly described and appropriate for the hypothesis being tested?

-Is the sample size sufficient to ensure adequate power to address the hypothesis being tested?

-Were correct statistical analysis used to support conclusions?

-Are there concerns about ethical or regulatory requirements being met?

Reviewer #1: Whit Editor

Reviewer #2: Minor re-organisation and additional information is needed in the Methodology section and specifics have been highlighted directly in the manuscript.

Reviewer #3: (No Response)

--------------------

**Results**

-Does the analysis presented match the analysis plan?

-Are the results clearly and completely presented?

-Are the figures (Tables, Images) of sufficient quality for clarity?

Reviewer #1: Whit Editor

Reviewer #2: The results are clearly presented including the Figures and Tables.

Reviewer #3: 1. Please add percent prevalence, and how it tracked among the 7 wild mammal species sampled. It would be helpful to add common names, and whether the species are carnivores, herbivores, or omnivores in a table format.

2. In the same table, it would be helpful to list the qPCR result, to gauge the success of the 15 locus typing schematic.

3. Publications studying T. gondii genotypes infecting wildlife samples have demonstrated that mixed strain T. gondii infections can occur, sometimes at high frequency, in the various target tissues examined herein – heterozygosity can dramatically impact consensus calling for SNPs and reak havoc on INDEL analyses, producing artefacts. What controls were run to obviate this concern.

4. Table 1 – is it possible to put a line that separates the two samples studied herein, from the 9 control samples that were added for comparative purposes.

5. Fig 2 – please add the location of the 16 reference genome assemblies to the AFC graphic

--------------------

**Conclusions**

-Are the conclusions supported by the data presented?

-Are the limitations of analysis clearly described?

-Do the authors discuss how these data can be helpful to advance our understanding of the topic under study?

-Is public health relevance addressed?

Reviewer #1: Whit Editor

Reviewer #2: Conclusions are well supported by the data and public relevance of the results and public health relevance is emphasized.

Reviewer #3: The conclusions are not supported by the data presented. Only one sample was studied at both multi-locus resolution, and at WGS, not a population. Further, insufficient depth of coverage was obtained to resolve the genome with high confidence. Did the authors map reads to the 15 locus typing markers - and did the WGS analysis support the allele designation resolved by PCR? Was there evidence in either the PCR or WGS analysis, for mixed strain superinfection of the tissue? To control for any potential artifact in the SNP calling pipelines to resolve the allele present. Much of the discussion centers on the Gabon strain being genetically related to the COUG isolate - but for the reasons above, this is circumspect, and not sufficiently supported, and the present study, in my opinion, does not provide evidence for the existence of a sylvatic cycle of T. gondii in Africa [lines 312-314]. Importantly, they do not provide evidence that an African wild T. gondii strain has emerged with a potential to cause severe toxoplasmosis among immunocompetent individuals - which has been demonstrated to occur in South and North America, but not herein.

--------------------

**Editorial and Data Presentation Modifications?**

Use this section for editorial suggestions as well as relatively minor modifications of existing data that would enhance clarity. If the only modifications needed are minor and/or editorial, you may wish to recommend “Minor Revision” or “Accept”. 

Reviewer #1: Whit Editor

Reviewer #2: I have recommended minor re-organisation of information to enhance clarity, and this is highlighted directly on the manuscript specifically in the methodology section.

Reviewer #3: (No Response)

--------------------

**Summary and General Comments**

Use this section to provide overall comments, discuss strengths/weaknesses of the study, novelty, significance, general execution and scholarship. You may also include additional comments for the author, including concerns about dual publication, research ethics, or publication ethics. If requesting major revision, please articulate the new experiments that are needed.

Reviewer #1: Whit Editor

Reviewer #2: The authors report on a study titled “Toxoplasma gondii from Gabonese forest, Central Africa: first report of an African wild

population”. I have carefully gone through the contents of the manuscript and the authors have rightfully justified the purpose of the study. The methodologies followed are sound and I have indicated my specific comments to enhance clarity directly on the manuscript for easy of revisions.

The study villages are only quantified without support of information on the geographical location of these in Gabon. My suggestion is for the authors to consider adding a map from their already published paper in this journal (Pierre Becquart et. al., 2024) which used the same study sites. This will assist the readership understanding without having to look for the publication with this information.

Reviewer #3: Toxoplasma gondii from Gabonese forest, Central Africa: first report of an African wild population

In this paper, 148 animals from at least seven species of wild mammals in Gabon Africa (collected as part of a viral screening program) were tested for the presence of Toxoplasma gondii (T. gondii) DNA by qPCR. T. gondii DNA was found in 15 animals from four different species that were collected from six villages, a prevalence of ~10%. The authors attempted to genotype the PCR positive samples using 15 typing markers that included a combination of 8 genotyping markers of limited variation and 7 fingerprinting microsatellite markers that are more varied across T. gondii lineages. Only one sample had sufficient DNA to yield a multi-locus genotype (at 12 of 15 markers). Two markers possessed new alleles, but it was not clear how divergent the new alleles were – whether they were different by a single SNP, an INDEL, or they were something more divergent. In the NJ tree, the Gabon-87 sample was distinct, and resolved on a single branch away from the majority of other MLGs, and it resolved closely with another isolate from a Sheep in Ethiopia. Is it known whether the animal infected with Gabon-87 was sick, and impacted by toxoplasmosis? Likewise for the Sheep from Ethiopia, as the paper discusses the relevance of the emergence of highly pathogenic strains from wildlife reservoirs. Using their published database of 1068 MLGs, a Factorial correspondence analysis (AFC) supported their divergent designation as both the sample from Ethiopia (not studied herein) and the Gabon-87 sample were distinct from the majority of other samples (Fig. 2) at the 12 loci examined. It would have been informative to have labeled Fig. 2 with the position of FOU (Africa 1), COUG, VEG and TgCtBr5, for example, the 4 reference genomes that had the most reads map from the Gabon-87 unenriched WGS sequencing effort. In an attempt to gather more T. gondii sequence from the infected tissue sample, a total of 1,371,024,686 paired-end illumina reads were obtained from the infected heart sample, of which 282,006 (representing 0.02% of trimmed reads) were T. gondii. This would represent about 84.6Mb of sequence assuming 300bp of high quality sequence was obtained for each paired read, or marginally 1X genome coverage. Not sure how they calculated an average depth of genome coverage at 3.5X [lines 217-218]? In a novel attempt to resolve genetic relatedness, the Gabon-87 reads were mapped against the 16 T. gondii reference genome assemblies at various mapping stringencies, and the majority of reads mapped, in descending order, to COUG, FOU (Africa 1), TgCtBr5 and VEG. The authors concluded that the Gabon-87 sample was more related to COUG than to the major domestic T. gondii lineages found in Gabon and Africa (type II, Africa 1, Africa 3) [lines 298-300], which I find difficult to support. While ~200K reads did map to the COUG assembly at -0.98 using smalt, ~100K reads mapped to the Africa 1 FOU assembly, ~80K reads to the TgCtBr5 assembly, and ~75K to the VEG (type III) assembly. Is this a novel recombinant? Did the mapped reads overlap for each assembly, or were they in separate parts of the genome? Clearly a significant number of reads are mapping to an Africa 1 isolate, which is a major domestic lineage circulating in Gabon. Hence, without enrichment, or an isolate, the authors can only infer the origin of the sample, and they only have data for one sample. However, the title suggests the existence of an African wild population of circulating T. gondii, but the analysis is for only one sample, so it is incomplete.

--------------------

PLOS authors have the option to publish the peer review history of their article (what does this mean? ). If published, this will include your full peer review and any attached files.

If you choose “no”, your identity will remain anonymous but your review may still be made public.

**Do you want your identity to be public for this peer review?** For information about this choice, including consent withdrawal, please see our Privacy Policy .

Reviewer #1: No

Reviewer #2: Yes: Samson Mukaratirwa

Reviewer #3: No

Figure Files:

While revising your submission, please upload your figure files to the Preflight Analysis and Conversion Engine (PACE) digital diagnostic tool, https://pacev2.apexcovantage.com. PACE helps ensure that figures meet PLOS requirements. To use PACE, you must first register as a user. Then, login and navigate to the UPLOAD tab, where you will find detailed instructions on how to use the tool. If you encounter any issues or have any questions when using PACE, please email us at figures@plos.org.

Data Requirements:

Please note that, as a condition of publication, PLOS' data policy requires that you make available all data used to draw the conclusions outlined in your manuscript. Data must be deposited in an appropriate repository, included within the body of the manuscript, or uploaded as supporting information. This includes all numerical values that were used to generate graphs, histograms etc.. For an example see here: http://www.plosbiology.org/article/info%3Adoi%2F10.1371%2Fjournal.pbio.1001908#s5.

Reproducibility:

To enhance the reproducibility of your results, we recommend that you deposit your laboratory protocols in protocols.io, where a protocol can be assigned its own identifier (DOI) such that it can be cited independently in the future. Additionally, PLOS ONE offers an option to publish peer-reviewed clinical study protocols. Read more information on sharing protocols at https://plos.org/protocols?utm_medium=editorial-email&utm_source=authorletters&utm_campaign=protocols

Attachment Submitted filename: PNTD-D-24-00685-Annotated.pdf

# Author comment: Author response to Decision Letter 0

**DOI:** 10.1371/journal.pntd.0012214.r002

25 Oct 2024

Attachment Submitted filename: responses_to_reviewers_Gabon_pop_sauvage_R1.docx

# Peer review: Decision Letter 1

**DOI:** 10.1371/journal.pntd.0012214.r003

**Authors:** Claudia Ida Brodskyn

12 Nov 2024

PNTD-D-24-00685R1Toxoplasma gondii from Gabonese forest, Central Africa: first report of an African wild populationPLOS Neglected Tropical Diseases Dear Dr. Fritz, Thank you for submitting your manuscript to PLOS Neglected Tropical Diseases. After careful consideration, we feel that it has merit but does not fully meet PLOS Neglected Tropical Diseases's publication criteria as it currently stands. Therefore, we invite you to submit a revised version of the manuscript that addresses the points raised during the review process. Please submit your revised manuscript within 30 days Dec 12 2024 11:59PM. If you will need more time than this to complete your revisions, please reply to this message or contact the journal office at plosntds@plos.org. When you're ready to submit your revision, log on to https://www.editorialmanager.com/pntd/ and select the 'Submissions Needing Revision' folder to locate your manuscript file. Please include the following items when submitting your revised manuscript:* A rebuttal letter that responds to each point raised by the editor and reviewer(s). You should upload this letter as a separate file labeled 'Response to Reviewers '. This file does not need to include responses to any formatting updates and technical items listed in the 'Journal Requirements' section below.* A marked-up copy of your manuscript that highlights changes made to the original version. You should upload this as a separate file labeled 'Revised Manuscript with Track Changes '.* An unmarked version of your revised paper without tracked changes. You should upload this as a separate file labeled 'Manuscript '. If you would like to make changes to your financial disclosure, competing interests statement, or data availability statement, please make these updates within the submission form at the time of resubmission. Guidelines for resubmitting your figure files are available below the reviewer comments at the end of this letter. We look forward to receiving your revised manuscript. Kind regards, Claudia Ida BrodskynSection EditorPLOS Neglected Tropical Diseases Claudia BrodskynSection EditorPLOS Neglected Tropical Diseases

Shaden Kamhawi

co-Editor-in-Chief

PLOS Neglected Tropical Diseases

orcid.org/0000-0003-4304-636XX

Paul Brindley

co-Editor-in-Chief

PLOS Neglected Tropical Diseases

orcid.org/0000-0003-1765-0002

 **Journal Requirements:** **Additional Editor Comments (if provided):**   [Note: HTML markup is below. Please do not edit.] **Reviewers' comments:** Reviewer's Responses to Questions

**Key Review Criteria Required for Acceptance?**

As you describe the new analyses required for acceptance, please consider the following:

**Methods**

-Are the objectives of the study clearly articulated with a clear testable hypothesis stated?

-Is the study design appropriate to address the stated objectives?

-Is the population clearly described and appropriate for the hypothesis being tested?

-Is the sample size sufficient to ensure adequate power to address the hypothesis being tested?

-Were correct statistical analysis used to support conclusions?

-Are there concerns about ethical or regulatory requirements being met?

Reviewer #4: This study is clear in aims and the results provide new clue information on Toxoplasma diversity

Reviewer #5: Overall the methods are sounds, objectives are clearly stated and addressed. Small number (N=2) of samples were successfully characterized, however this weakness is addressed by the authors.

Reviewer #6: -Are the objectives of the study clearly articulated with a clear testable hypothesis stated?

YES

-Is the study design appropriate to address the stated objectives?

YES

-Is the population clearly described and appropriate for the hypothesis being tested?

YES

-Is the sample size sufficient to ensure adequate power to address the hypothesis being tested?

Detaileed data on a single isolate only appears not enough to conclude on a wild population.

-Were correct statistical analysis used to support conclusions?

Not applicable.

-Are there concerns about ethical or regulatory requirements being met?

NO

**********

**Results**

-Does the analysis presented match the analysis plan?

-Are the results clearly and completely presented?

-Are the figures (Tables, Images) of sufficient quality for clarity?

Reviewer #4: The information was obtained with methods that are adequate for the purpose of the study and figures are informative

Reviewer #5: The analysis is sound and most of the figures are clearly provided. One figure lacks axes labels (noted in comments).

Reviewer #6: -Does the analysis presented match the analysis plan?

YES

-Are the results clearly and completely presented?

YES

-Are the figures (Tables, Images) of sufficient quality for clarity?

Figure 4 and Supplementary Figures need axes descriptions. Furthermore I am a bit puzzled - There is a Fig4 in the text and a Fig4ab after the text, i.e. there are two different Fig. 4. ???

**********

**Conclusions**

-Are the conclusions supported by the data presented?

-Are the limitations of analysis clearly described?

-Do the authors discuss how these data can be helpful to advance our understanding of the topic under study?

-Is public health relevance addressed?

Reviewer #4: The discussion is well suported

Reviewer #5: The limitation of sample size is acknowledged by the authors. A major gap in knowledge on the global epidemiology of Toxoplasma is lack of studies in Africa, which this paper strives to address. The authors note that additional studies will be needed for more clarity on population genetics of the parasite in this continent, as well as to understand whether certain strains are associated with more virulent disease in this region.

Reviewer #6: -Are the conclusions supported by the data presented?

Line 1 -2 "population" goes too far, to my opinion. I suggest the following title or similar: "A unique Toxoplasma gondii genotype from Gabonese forest suggests the excistence of an African wild toxoplasma population "

Line 305: It is impossible for me to accept the use of "T. gondii population" if only a single representative (i.e. a single isolate) has been identified yet. I suggest to rephrase. Couldn't this single isolate be the result of sexual recombination of representatives of true T. gondii populations?

-Are the limitations of analysis clearly described?

YES

-Do the authors discuss how these data can be helpful to advance our understanding of the topic under study?

YES

-Is public health relevance addressed?

YES

**********

**Editorial and Data Presentation Modifications?**

Use this section for editorial suggestions as well as relatively minor modifications of existing data that would enhance clarity. If the only modifications needed are minor and/or editorial, you may wish to recommend “Minor Revision” or “Accept”.

Reviewer #4: No suggestions

Reviewer #5: See notes in the next section

Reviewer #6: Further suggestions:

Line 59: "may mark" istead of "mark"

Line 74: "may mark" insrtead of "mark"

There is no definition of "Wild strain" given in the introduction. Please add. Some examples on "wild strains" or "wild polulations" might also improve introduction.

Line 128: Explain abbreviation "qPCR" already here and not in Line 139.

Line 161: You had no results for 3 of the typing marker regions M33, IV.1 and XI.1. How did you treat this missing information when generating n-j-dendrogram?

Line 182: Please intoduce the abbreviation for "haplogroup" here.

Line 184: Please use only the abbreviation "hg" here.

Line 185: Please replace "haplogroup" by "hg"

Line 219: Please write "...M102, a fingerprinting marker". This is important because new alleles in finger printing markers are not such a surprise.

Line 257: What is the meaning of "uniquely mapping"?

Did these about 70 reads map exclusively to TgCtPRC2 ? Why are there two versions of Figure 4?

**********

**Summary and General Comments**

Use this section to provide overall comments, discuss strengths/weaknesses of the study, novelty, significance, general execution and scholarship. You may also include additional comments for the author, including concerns about dual publication, research ethics, or publication ethics. If requesting major revision, please articulate the new experiments that are needed.

Reviewer #4: The authors described the presence, by PCR of Toxoplasma in wild species in Gabon (Africa). They sequenced one new complete genome. The descriptions add valuable information. The manuscript was well written. I have made some minor remarks.

- Please include the percentage of positive results by species. The reader can calculate this, but it can ease the lecture if percentages are shown. Please think of the readers.

- Why do authors not estimate the molecular clock? I think it is important if any estimates between the strains are assayed by calibrating with genes as done in Plasmodium: Science. 2010;329(5988):226-9. doi: 10.1126/science.1188954

- Please include the database where the complete genome sequence was submitted and the accesion number.

Reviewer #5: This is a well written and timely manuscript on circulating genomic strains of Toxoplasma gondii in Gabon, Africa – a region vastly understudied for this parasite. The authors describe application of both an established (MS) as well as advanced (WGS) approach to evaluate genetic diversity of T. gondii strains. The scarcity of samples that contained high enough levels of parasite DNA for characterization is a weakness that was noted by the authors, as is the need to further evaluate whether ‘wild’ strains in Africa are associated with more severe toxoplasmosis in humans and animals. I have provided a few comments and edits for the authors’ consideration. Overall, this paper provides an important advance to the field of Toxoplasma epidemiology, especially given lack of studies on this globally important pathogen from the continent of Africa.

Specific comments:

L48 - Compared with other globally prevalent pathogens, and even related apicomplexans (e.g. Sarcocystis spp), T. gondii is genetically quite conserved and rather limited in diversity, though this perception may depend on the context; this is just a note, not necessarily requiring authors to edit

L60 - Could edit here and elsewhere from ‘population’ to 'strain' to address concerns from other reviewers that the lack of genetic characterization on more than two samples (or one for WGS) does not justify the term 'population' in this context

L168 - Describe what you mean by 'high' (e.g. what Ct value exactly)?

L198 - Were positive qPCR results from screening assay confirmed via sequencing? Since the 529 RE target, while often described as Toxo-specific, can lead to non-specific amplification as well.

L215 - What is typically considered low enough Ct to yield positive MS typing? It might be interesting to present the Ct results for the positive samples (as sup data). Was the successfully typed samples (N=2) notably lower Ct than other samples for which MS typing was not successful?

L 215 – here and general question/comment – I’m curious as to whether the authors considered the nested PCR MLST approach commonly used in North America for genetically characterizing the qPCR-positive samples? the nested PCR approach may be more sensitive than MS typing for DNA characterization in tissues with low abundance of Tg DNA. The authors may wish to comment on the choice for genotyping approaches in the discussion, and other options that may allow for characterization of such samples (even if at lower resolution than MS typing).

Figures:

In-text Fig 4. Please add Y and X axes labels

Fig 4 with two panels on P. 25: Is this figure supposed to be in main paper? Above for Fig 4 within text I only see one panel for a similar figure

- And please add labels for axes. unclear if these are number of reads?

Reviewer #6: This represents an interesting study providing an indication that there is more genotypic variation in Toxoplasma gondii in Africa than known yet.

**********

PLOS authors have the option to publish the peer review history of their article (what does this mean? ). If published, this will include your full peer review and any attached files.

If you choose “no”, your identity will remain anonymous but your review may still be made public.

**Do you want your identity to be public for this peer review?** For information about this choice, including consent withdrawal, please see our Privacy Policy .

Reviewer #4: No

Reviewer #5: **Yes: ** Karen Shapiro

Reviewer #6: No

 [NOTE: If reviewer comments were submitted as an attachment file, they will be attached to this email and accessible via the submission site. Please log into your account, locate the manuscript record, and check for the action link "View Attachments". If this link does not appear, there are no attachment files.] **Figure resubmission:** While revising your submission, please upload your figure files to the Preflight Analysis and Conversion Engine (PACE) digital diagnostic tool, https://pacev2.apexcovantage.com/. PACE helps ensure that figures meet PLOS requirements. To use PACE, you must first register as a user. Registration is free. Then, login and navigate to the UPLOAD tab, where you will find detailed instructions on how to use the tool. If you encounter any issues or have any questions when using PACE, please email PLOS at figures@plos.org. Please note that Supporting Information files do not need this step. If there are other versions of figure files still present in your submission file inventory at resubmission, please replace them with the PACE-processed versions. **Reproducibility:** To enhance the reproducibility of your results, we recommend that authors of applicable studies deposit laboratory protocols in protocols.io, where a protocol can be assigned its own identifier (DOI) such that it can be cited independently in the future. Additionally, PLOS ONE offers an option to publish peer-reviewed clinical study protocols. Read more information on sharing protocols at https://plos.org/protocols?utm_medium=editorial-email&utm_source=authorletters&utm_campaign=protocols

# Author comment: Author response to Decision Letter 1

**DOI:** 10.1371/journal.pntd.0012214.r004

29 Nov 2024

Attachment Submitted filename: 20241113_PNTD_responses_to_reviewers_R2_final.docx

# Peer review: Decision Letter 2

**DOI:** 10.1371/journal.pntd.0012214.r005

**Authors:** Claudia Ida Brodskyn

17 Dec 2024

Dear Dr. Fritz,

We are pleased to inform you that your manuscript 'Toxoplasma gondii from Gabonese forest, Central Africa: first report of an African wild strain' has been provisionally accepted for publication in PLOS Neglected Tropical Diseases.

Before your manuscript can be formally accepted you will need to complete some formatting changes, which you will receive in a follow up email. A member of our team will be in touch with a set of requests.

Please note that your manuscript will not be scheduled for publication until you have made the required changes, so a swift response is appreciated.

IMPORTANT: The editorial review process is now complete. PLOS will only permit corrections to spelling, formatting or significant scientific errors from this point onwards. Requests for major changes, or any which affect the scientific understanding of your work, will cause delays to the publication date of your manuscript.

Should you, your institution's press office or the journal office choose to press release your paper, you will automatically be opted out of early publication. We ask that you notify us now if you or your institution is planning to press release the article. All press must be co-ordinated with PLOS.

Thank you again for supporting Open Access publishing; we are looking forward to publishing your work in PLOS Neglected Tropical Diseases.

Best regards,

Claudia Ida Brodskyn

Section Editor

PLOS Neglected Tropical Diseases

Claudia Brodskyn

Section Editor

PLOS Neglected Tropical Diseases

Shaden Kamhawi

co-Editor-in-Chief

PLOS Neglected Tropical Diseases

orcid.org/0000-0003-4304-636XX

Paul Brindley

co-Editor-in-Chief

PLOS Neglected Tropical Diseases

orcid.org/0000-0003-1765-0002

***********************************************************

Reviewer's Responses to Questions

**Key Review Criteria Required for Acceptance?**

As you describe the new analyses required for acceptance, please consider the following:

**Methods**

-Are the objectives of the study clearly articulated with a clear testable hypothesis stated?

-Is the study design appropriate to address the stated objectives?

-Is the population clearly described and appropriate for the hypothesis being tested?

-Is the sample size sufficient to ensure adequate power to address the hypothesis being tested?

-Were correct statistical analysis used to support conclusions?

-Are there concerns about ethical or regulatory requirements being met?

Reviewer #4: The objectives, methosd and limitation of the study are clearly presented and discussed.

Reviewer #5: Methods are sound, my previous questions regarding the methods were addressed by the authors. I have no additional comments here.

Reviewer #6: (No Response)

**********

**Results**

-Does the analysis presented match the analysis plan?

-Are the results clearly and completely presented?

-Are the figures (Tables, Images) of sufficient quality for clarity?

Reviewer #4: The results correspond with the analysis plan

Reviewer #5: The results are sound. I only have one minor question for the authors Re. 529 RE PCR specificity. In their response to a previous question, the authors note that they added the following sentences: “In addition, the molecular prevalence levels may have been slightly underestimated due to the possible weak cross-reactivity of the primers targeting the T. gondii 529 bp repeat region with Hammondia DNA, as previously reported by Schares et al. (2008).”

Please clarify if you meant to state ‘overestimated’ vs ‘underestimated’? i.e. given potential for ‘false positives’ due to cross-reactivity of the assay with Hammondia detection, one would assume including potential Hammondia infections within the reported prevalences for Toxo would be an overestimation vs an underestimation?

Reviewer #6: (No Response)

**********

**Conclusions**

-Are the conclusions supported by the data presented?

-Are the limitations of analysis clearly described?

-Do the authors discuss how these data can be helpful to advance our understanding of the topic under study?

-Is public health relevance addressed?

Reviewer #4: The discussion undertakes the current knowledge and challenges of typing Toxoplasma in wild environment. It give a clear picture for reader of what is the contribution of present description

Reviewer #5: The conclusions are supported by the data and limitations are explained by the authors. The study explains how the results advance our understanding of Toxoplasma molecular diversity in Gabon, Africa.

Reviewer #6: (No Response)

**********

**Editorial and Data Presentation Modifications?**

Use this section for editorial suggestions as well as relatively minor modifications of existing data that would enhance clarity. If the only modifications needed are minor and/or editorial, you may wish to recommend “Minor Revision” or “Accept”.

Reviewer #4: Accept

Reviewer #5: My previous recommendations for clarifying Figure 4 were addressed, I have no additional comments.

Reviewer #6: (No Response)

**********

**Summary and General Comments**

Use this section to provide overall comments, discuss strengths/weaknesses of the study, novelty, significance, general execution and scholarship. You may also include additional comments for the author, including concerns about dual publication, research ethics, or publication ethics. If requesting major revision, please articulate the new experiments that are needed.

Reviewer #4: The authors answered the questions satisfactorily. I found the percentage of prevalence by species in Supplemental Table 2. Thank you for pointing this out to us. Also, it is clear now, for me, that there are difficulties in calculating the molecular clock. The presentation of results has been improved by following the questions from other reviewers, and I think now the interpretation of the results is clearer.

Reviewer #5: I have no further comments and believe this paper provides an important contribution to our understanding of global Toxoplasma epidemiology and molecular diversity.

Reviewer #6: (No Response)

**********

PLOS authors have the option to publish the peer review history of their article (what does this mean? ). If published, this will include your full peer review and any attached files.

If you choose “no”, your identity will remain anonymous but your review may still be made public.

**Do you want your identity to be public for this peer review?** For information about this choice, including consent withdrawal, please see our Privacy Policy .

Reviewer #4: No

Reviewer #5: **Yes: ** Karen Shapiro

Reviewer #6: No
